# Dual-peptide engineered macrophage membrane biomimetic nanosystem via targeting Rg1 delivery for traumatic brain injury therapy

**DOI:** 10.1093/rb/rbag059

**Published:** 2026-03-20

**Authors:** Weiquan Liao, Zhichao Lu, Ziheng Li, Chenxing Wang, Xingjia Zhu, Jue Zhu, Yongqi Zhu, Jialiang Lin, Jiajia Wen, Xuanfeng Chen, Jian Chen, Jianhong Shen, Youlang Zhou, Peipei Gong

**Affiliations:** Department of Neurosurgery, Affiliated Hospital of Nantong University, Medical School of Nantong University, Nantong, Jiangsu 226001, China; Research Center of Clinical Medicine, Affiliated Hospital of Nantong University, Nantong, Jiangsu 226001, China; Department of Neurosurgery, Affiliated Hospital of Nantong University, Medical School of Nantong University, Nantong, Jiangsu 226001, China; Research Center of Clinical Medicine, Affiliated Hospital of Nantong University, Nantong, Jiangsu 226001, China; Department of Neurosurgery, Affiliated Hospital of Nantong University, Medical School of Nantong University, Nantong, Jiangsu 226001, China; Research Center of Clinical Medicine, Affiliated Hospital of Nantong University, Nantong, Jiangsu 226001, China; Department of Neurosurgery, Affiliated Hospital of Nantong University, Medical School of Nantong University, Nantong, Jiangsu 226001, China; Research Center of Clinical Medicine, Affiliated Hospital of Nantong University, Nantong, Jiangsu 226001, China; Department of Neurosurgery, Affiliated Hospital of Nantong University, Medical School of Nantong University, Nantong, Jiangsu 226001, China; Research Center of Clinical Medicine, Affiliated Hospital of Nantong University, Nantong, Jiangsu 226001, China; Department of Neurosurgery, Affiliated Hospital of Nantong University, Medical School of Nantong University, Nantong, Jiangsu 226001, China; Research Center of Clinical Medicine, Affiliated Hospital of Nantong University, Nantong, Jiangsu 226001, China; Department of Neurosurgery, Affiliated Hospital of Nantong University, Medical School of Nantong University, Nantong, Jiangsu 226001, China; Research Center of Clinical Medicine, Affiliated Hospital of Nantong University, Nantong, Jiangsu 226001, China; Department of Neurosurgery, Affiliated Hospital of Nantong University, Medical School of Nantong University, Nantong, Jiangsu 226001, China; Research Center of Clinical Medicine, Affiliated Hospital of Nantong University, Nantong, Jiangsu 226001, China; Department of Neurosurgery, Affiliated Hospital of Nantong University, Medical School of Nantong University, Nantong, Jiangsu 226001, China; Research Center of Clinical Medicine, Affiliated Hospital of Nantong University, Nantong, Jiangsu 226001, China; Department of Neurosurgery, Affiliated Hospital of Nantong University, Medical School of Nantong University, Nantong, Jiangsu 226001, China; Research Center of Clinical Medicine, Affiliated Hospital of Nantong University, Nantong, Jiangsu 226001, China; Department of Neurosurgery, Affiliated Hospital of Nantong University, Medical School of Nantong University, Nantong, Jiangsu 226001, China; Department of Neurosurgery, Affiliated Hospital of Nantong University, Medical School of Nantong University, Nantong, Jiangsu 226001, China; Research Center of Clinical Medicine, Affiliated Hospital of Nantong University, Nantong, Jiangsu 226001, China; Department of Neurosurgery, Affiliated Hospital of Nantong University, Medical School of Nantong University, Nantong, Jiangsu 226001, China; Jiangsu Medical Innovation Center, Neurological Disease Diagnosis and Treatment Center, Affiliated Hospital of Nantong University, Nantong, Jiangsu 226001, China

**Keywords:** traumatic brain injury, Ginsenoside Rg1, cell membrane, astrocytes, transdifferentiation

## Abstract

Traumatic brain injury (TBI) induces a detrimental inflammatory microenvironment at the lesion site, which, together with neuronal death and loss, leads to neurological dysfunction. The blood–brain barrier (BBB) further impedes intracerebral drug delivery, posing a major challenge for post-TBI therapy. To overcome this, we developed a brain-targeted biomimetic nanosystem (R/T-MaM-NPs) using an engineered dual-peptide-modified macrophage membrane (MaM). This system encapsulates neuroprotective ginsenoside Rg1 into poly (lactic-co-glycolic acid)-based nanoparticles (NPs). RAW264.7 macrophages were engineered to co-express targeting peptides (RVG and T7) on their membranes; the derived R/T-MaM was then coated onto NPs. The MaM coating conferred high biocompatibility and biosafety, enabling R/T-MaM-NPs to reduce immune clearance and prolong systemic circulation. By leveraging the intrinsic inflammatory chemotaxis of MaM and dual-peptide targeting, the integrated system promoted traversal across the BBB and subsequent accumulation around the cerebral lesion, thereby inducing the transdifferentiation of reactive astrocytes (RAs) into electrophysiologically functional neuron-like cells. RNA sequencing confirmed significant upregulation of neurogenic genes in R/T-MaM-NP-treated RAs, an outcome closely linked to suppression of the Wnt/Notch signaling pathway. Furthermore, R/T-MaM-NPs remodeled the inflammatory microenvironment at the TBI site, alleviated cerebral edema, and enhanced the recovery of cognitive and motor functions in TBI mice.

## Graphical Abstract

**Graphical Abstract:**
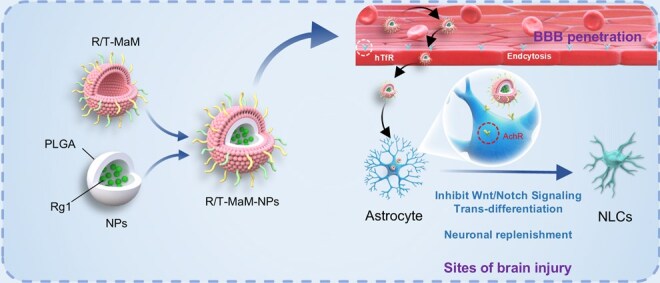

## Introduction

Traumatic brain injury (TBI) is defined as brain damage induced by external mechanical forces, representing the most prevalent form of clinical central nervous system (CNS) injury [1]. Based on distinct morphological and pathological characteristics post-injury, TBI is generally classified into focal and diffuse subtypes. The pathological cascade encompasses axonal shearing, cellular edema, vascular disruption, and neuronal death [2]. The limited regenerative capacity of the mature CNS arises from two fundamental constraints: (1) intrinsically restricted regenerative potential of mature neurons and (2) post-injury microenvironmental hostility to neural regeneration in the adult CNS [3, 4]. Consequently, strategic enhancement of *de novo* neurogenesis coupled with inflammatory microenvironmental remodeling constitutes critical therapeutic targets for post-TBI functional recovery. Although stem cell-based therapies hold promise for neural circuit preservation, practical implementation faces substantial challenges including narrow therapeutic temporal windows, donor cell source limitations, safety concerns, and immune rejection risks [5, 6].

Astrocytes play pivotal roles in the CNS by supporting neuronal function through synaptic facilitation, structural plasticity, and homeostatic maintenance [7]. However, following CNS injury, these cells are rapidly activated into reactive astrocytes (RAs) that proliferate to form glial scars, creating a major physical and biochemical barrier to neuronal regeneration [8]. Recent studies have demonstrated that reprogramming astrocytic gene expression to induce neurogenesis-associated proteins (e.g. Notch, Sox2, Neurod1, Shh, Ascl1, Brn2) enables direct *in vitro* conversion of astrocytes into functionally competent neuron-like cells (NLCs) [9, 10]. This breakthrough provides a viable strategy for generating replacement neurons at TBI lesion sites, which not only facilitates *de novo* neurogenesis but also mitigates the inhibitory effects of reactive astrogliosis on tissue repair processes [11].

Ginsenoside Rg1 (Rg1) exhibits multifaceted therapeutic effects in cardiovascular, immune, and nervous system protection [12, 13]. Mechanistically, Rg1 promotes astrocytic transdifferentiation by upregulating neurogenesis-associated proteins, thereby facilitating neuronal repair, suppressing apoptosis, and enhancing axonal regeneration [14, 15]. Therefore, Rg1 holds therapeutic potential for post-TBI neuronal replenishment through targeted astrocyte reprogramming. However, a major challenge in CNS therapeutics lies in overcoming the blood–brain barrier (BBB). The BBB excludes >98% of systemically administered compounds from entering the brain parenchyma, with only trace amounts reaching the CNS at subtherapeutic concentrations. This profoundly limits targeted drug delivery to TBI lesion areas [16, 17].

Notably, biomimetic membrane-coated nanoparticles (NPs) are increasingly being utilized in CNS disorder therapeutics due to their intrinsic biological interfaces, superior biocompatibility, and biosafety profile. These nanocarriers enhance BBB permeability through biomimetic transport mechanisms, enabling improved cerebral bioavailability of therapeutic agents with reduced systemic exposure [18, 19]. The injury site post-TBI is characterized by an inflammatory storm microenvironment [20]. Interestingly, macrophages (Ma), as pivotal immune cells *in vivo*, possess an innate ability to migrate toward inflammatory regions in the body [21]. This innate inflammatory-oriented chemotactic capability facilitates the accumulation of drug-loaded macrophages in the acutely inflamed brain following TBI. Macrophage polarization plays critical roles in pathogen defense, inflammation modulation, tissue repair, and homeostatic maintenance. Specifically, M2-type macrophages mediate anti-inflammatory responses and Th2 immune polarization, functioning to suppress inflammation and promote tissue repair [22, 23]. Thus, leveraging this biological property, we employed interleukin (IL)-4-polarized M2 MaMs to camouflage therapeutics for brain-targeted drug delivery [24, 25].

**Figure 1:**
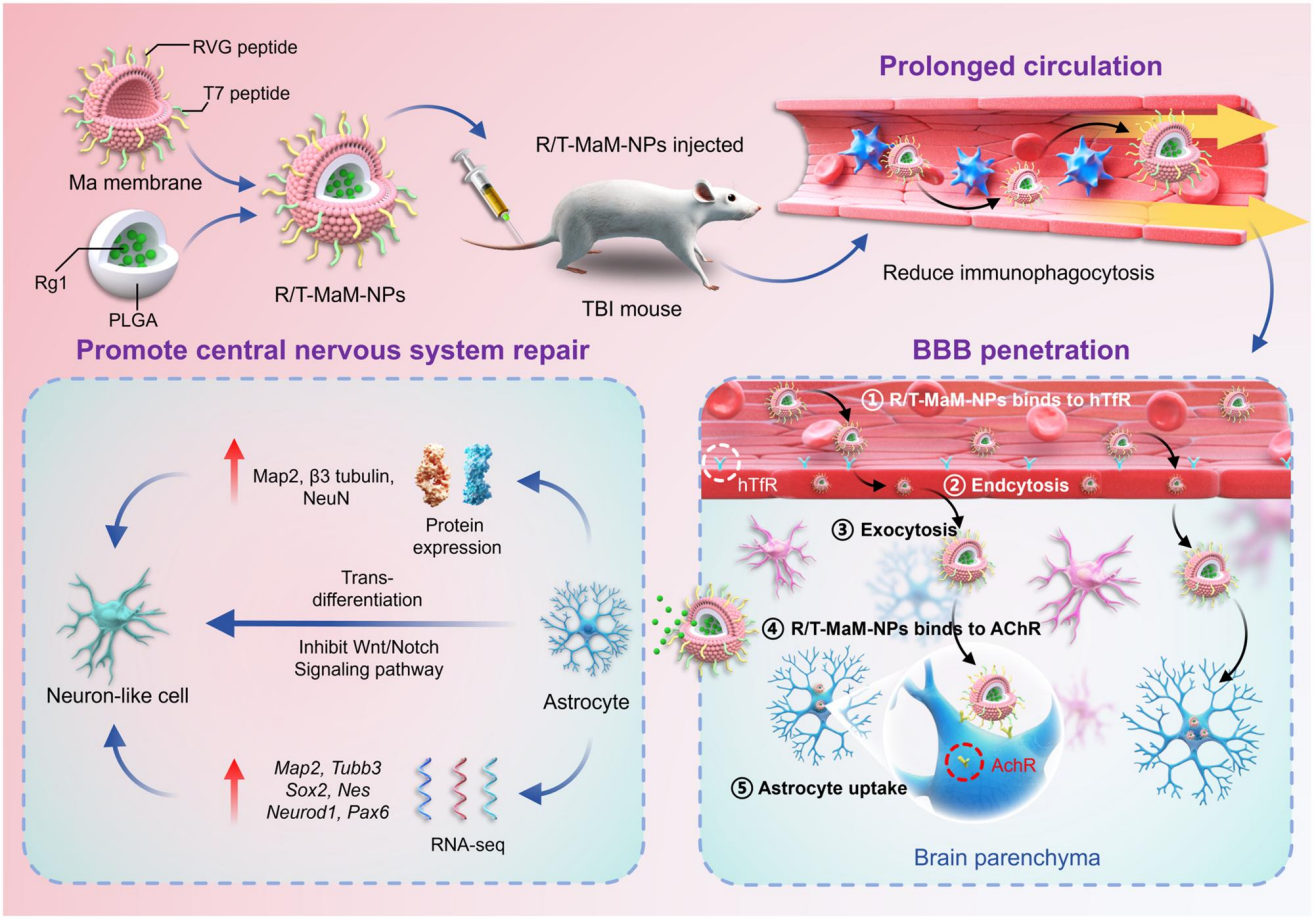
Schematic of genetically engineered macrophage membrane-coated nanoformulations (R/T-MaM-NPs) and their application in drug delivery to the CNS.

In this study, we engineered a biomimetic nanosystem (R/T-MaM-NPs) coated with genetically modified MaM (Figure 1). The MaMs were dual-functionalized via lentiviral transfection with RVG and T7 peptides: (1) RVG peptide targets acetylcholine receptors (AChRs) on neural cells, including astrocytes [26, 27], while (2) T7 exhibits high binding affinity for transferrin receptors (TfRs) at the BBB [28, 29]. This dual-peptide modification enables the ginsenoside Rg1-loaded NPs to cross the BBB and reach the brain lesion sites, promoting their uptake by RAs and inducing their transdifferentiation. Simultaneously, the fluorescence-labeled NPs allow for real-time visualization of their biodistribution and brain-targeting efficacy both *in vitro* and *in vivo*.

Results demonstrate seven key advantages of our engineered nanosystem: (i) inherent inflammatory chemotaxis of MaM drives brain-targeted drug delivery after TBI [30]; (ii) MaM-camouflaged architecture ensures enhanced biocompatibility and biosafety and prolongs blood circulation time while evading immune clearance; (iii) dual-peptide functionalization synergistically enhances BBB penetration capacity and promotes the uptake of nanosystems into astrocytes at TBI lesions; (iv) R/T-MaM-NPs induce astrocytic expression of neurogenesis-associated proteins, enabling transdifferentiation into electrophysiologically functional NLCs; (v) anti-inflammatory modulation suppresses neurotoxic cytokine release and inhibits lesion-associated apoptosis of cells; (vi) R/T-MaM-NPs ameliorated cerebral edema and reduced lesion size in mice following TBI; and (vii) functional restoration significantly improves post-TBI neurocognitive performance in spatial learning and motor coordination.

## Materials and methods

### Preparation of lentivirus

#### Plasmid construction

The gene (SP-RVG-TM-FLAG-FT2A-SP-T7-TM-MYC) was obtained from the cDNA library of Genechem (Shanghai, China) using the following amplification primers: The gene forward: 5′-CCGGATCCCCGGGTACCGGTCGCCACCATGGAGACACACACTCCTGCTATG-3′ and reverse: 5′‐AACTGACACACTTAGTCGACTCACAGATCCTCCTCTGAGATCAGCTTC-3′.

The vector GV656 (pRRLSIN-cPPT-SFFV-MCS-SV40-puromycin) (purchased from Shanghai Gene Chemical Co., Ltd.) was digested with AgeI and SalI restriction enzymes and recombined and ligated with the digested vector by adding complementary paired bases at both ends of the target sequence. Amplification products were recombinantly ligated to vectors digested by restriction endonuclease, and the correct sequence was determined by polymerase chain reaction (PCR) amplification and DNA sequencing. Lentiviral production was performed using Lipofectamine 2000 (Invitrogen; Thermo Fisher Scientific, Inc.) and two helper plasmids, psPAX2 and pMD2.G. Infectious lentiviruses were harvested 72 h after transfection, rapidly centrifuged to remove cellular debris, and then filtered through 0.45-μm cellulose acetate filters. Viral titer was determined by quantitative PCR analysis at approximately 1 × 10^9^ transduction units (TU)/mL of medium and stored at −80°C for further use.

### RAW264.7 transduction

RAW264.7 cells were infected using prepared lentiviruses to express overexpression of RVG and T7 double peptides on their cell membranes as described previously. RAW264.7 cells were inoculated in a six-well cell culture plate at a density of 5 × 10^4^ cells/mL for 24 h. The appropriate amount of virus was then added, and the medium was replaced with a new one after 72 h. Next, uninfected cells were removed with the appropriate concentration of puromycin, and RAW264.7 cells that had been transfected were obtained. The transduction efficiency of GFP-expressing control lentivirus in RAW264.7 cells was analyzed by flow cytometry.

### M2 polarization and identification of RAW264.7 cells

Transfected RAW264.7 cells were stimulated with 10 ng/mL IL-4 (Peprotech) for 24 h and subsequently harvested. The cell suspension was stained with fluorochrome-conjugated antibodies for 30 min on ice. After staining, cells were washed and resuspended in FACS buffer. M2 macrophage markers were then detected using a FACS Aria III flow cytometer, and the acquired data were analyzed with FlowJo software. Antibody details are provided in Supplementary Table S1.

### Cell culture

Referring to the literature [31], primary astrocytes were isolated from the hippocampus of Institute for Cancer Research (ICR) mice (1 day). The cells were seeded at a density of 1.0 × 10^5^/cm^2^ in a poly-l-lysine-coated T75 culture bottle and cultured in complete medium supplemented with 10% CO_2_ (DMEM + 10% fetal bovine serum + 1% penicillin/streptomycin). After mixing, the cells were placed in an incubator at 37°C with 5% CO_2_.

### Cell membrane extraction

Cultivate RAW264.7 cells expressing RVG and T7 peptides in large quantities, remove the complete medium, and add an appropriate amount of PBS pre-cooled in an ice bath. Use a cell scraper to scrape the cells from the bottom of the culture dish and centrifuge (20°C, 1000 rpm, 5 min) to form a pellet. Discard the supernatant and aspirate the remaining liquid as best as possible. Add a suitable amount of membrane protein extraction reagent A (with 1% PMSF, Beyotime, China) to the remaining cell mass, gently resuspend the cells, and place them in an ice box (4°C, 15 min). Then, use ultrasound to break the cells, centrifuge (4°C, 700 g, 10 min), carefully collect the supernatant in a new centrifuge tube, and remove unbroken cells and nuclei. The liquid in the new centrifuge tube was then centrifuged (4°C, 14 000 g, 30 min), and the precipitate, consisting of cell membrane fragments, was stored at −80°C for further use.

### Western blot

Tissues (RAs were isolated from the tissues by flow cytometry sorting) or RAs cell clusters were thoroughly mixed with an appropriate amount of cell lysis buffer (1% protease inhibitor and 1% phosphatase inhibitor), and the cells were broken up by sonication. After standing for 40 min, the mixture was centrifuged (12 000 g, 15 min) and the supernatant collected. All of the above operations were performed on ice. Total protein concentration using the BCA kit. The protein was mixed well with the loading buffer and boiled. Gel electrophoresis [sodium dodecyl sulfate-polyacrylamide gel electrophoresis (SDS-PAGE)] was then performed on a 10% gel to effectively separate proteins of different molecular weights. The proteins were then transferred to a polyvinylidene fluoride (PVDF) membrane. After blocking with 5% bovine serum albumin solution for 2 h, the PVDF membrane was incubated overnight with the primary antibody. The PVDF membrane was then incubated with the secondary antibody at room temperature for 2 h, and the protein bands were observed using ECL (Billerica Millipore, USA). (Bio-Rad, USA) and analyzed using ImageJ software. Specific details of the antibodies used can be found in Supplementary Table S1.

### Synthesis and characterization of NPs

#### Preparation of drug-encapsulated NPs

NPs containing Ginsenoside Rg1 (S3923, Selleck) were prepared using the liquid-solvent evaporation technique. A 200 μL of DMSO solution containing 10 mg of Rg1 or 10 mg of FITC (3326-32-7, MCE)/DiR (100068-60-8, MCE)/DiD (127274-91-3, MCE) was added to 1 mL of dichloromethane containing 100 mg of PLGA (lactic acid-to-glycolic acid ratio 65:35, molecular weight = 40 000–75 000, Sigma-Aldrich), and then incubated for 0.5 min in an ice bath using an ultrasonic homogenizer (Sonoplus HD 2070, Bandelin Electronic, Berlin, Germany). Then, the NPs were collected and resuspended in deionized water, and the above extracted MaM was doped into the NPs solution at a mass ratio of 1:2 (NPs:MaM) to ensure that an excess of MaM encapsulated the NPs. After sonication for 10 min, the NPs were then centrifuged (10 000 rpm for 5 min), and the precipitate was resuspended in equal volumes. Finally, MaM-NPs were extruded and obtained using a nanomaterial extruder.

Transmission electron microscopy (TEM) analysis was performed using an HT7800/HT7700 system (Hitachi, Japan). The hydrodynamic diameter and zeta potential of NPs were measured by dynamic light scattering (DLS). UV-Vis absorbance of Rg1 and R/T-MaM-NPs was measured using a UV–visible spectrophotometer (Shimadzu, Japan) at wavelengths ranging from 200 to 450 nm. SDS-PAGE was employed to analyze protein profiles of MaM, NPs, MaM-NPs, and R/T-MaM-NPs. All experiments were performed in triplicate.

### Coating efficiency of R/T-MaM-NPs

NPs and MaMs were labeled with FITC and PKH26 dyes, respectively (resulting in green NPs-FITC and red R/T-MaM-PKH26). R/T-MaM-NPs were prepared using formulations with varying ratios (NPs: R/T-MaM). All experiments were performed in triplicate. The coating efficiency of R/T-MaM-NPs was assessed by analyzing dual-color colocalization using ImageJ software.

### Loading and release of Rg1

The supernatant collected after centrifugation during R/T-MaM-NPs synthesis was subjected to UV-Vis spectroscopic analysis. Absorbance at 208 nm was measured to calculate the drug-loading (DL) efficiency and encapsulation efficiency (EE) of Rg1 using a pre-established standard calibration curve. Subsequently, the NPs were loaded into pre-hydrated dialysis bags immersed in separate beakers containing 10 mL of PBS (pH 7.4). The system was maintained at 37°C under continuous agitation (100 rpm) in a thermostatic shaker. At predetermined intervals, 2-mL aliquots were withdrawn and replaced with an equal volume of fresh PBS under identical temperature and pH conditions. The cumulative release of Rg1 at each time point was quantified using UV-Vis spectroscopy at 208 nm. All drug release experiments were performed in triplicate (*n* = 3). The DL and EE of R/T-MaM-NPs were calculated using the following formulas (1) and (2):

1. DL (%) = (mass of drug encapsulated in NPs/total mass of NPs) × 100%
2. EE (%) = (mass of drug encapsulated in NPs/total mass of drug fed initially) × 100%

### Cytotoxicity test

Primary astrocytes, Bend.3 cells, RAW264.7 cells, Bv2 cells, and Ht22 cells were cultured separately in 96-well plates containing 1 × 10^4^ cells per well (100 μL, Pricella, China) and incubated overnight. Equal concentrations of Rg1, NPs, MaM-NPs, and R/T-MaM-NPs (40 μg/ml) were added after replacing the medium. After culturing for 12, 24, or 36 h, CCK-8 (10 μL/well) (C0042, Beyotime) was added at different time points, and the absorbance at 450 nm was read using a multi-mode microplate reader after incubation at 37°C for 2 h. All experiments were performed in triplicate.

### Plasma stability evaluation of NPs

Following extrusion using a nanomaterial extruder, the plasma stability was assessed by mixing the NPs (NPs and R/T‑MaM‑NPs at a concentration of 2 mg/mL) with an equal volume of fresh mouse plasma. The mixture was incubated in a constant temperature water bath at 37°C under continued stirring to simulate the *in vivo* environment. At designated time points (0, 2, 4, 8, 12, and 24 h), 1 mL aliquots were withdrawn and subjected to ultracentrifugation (4°C, 100 000 g, 30 min). The pelleted NPs were resuspended in fresh PBS and washed twice, and their hydrodynamic diameter was measured by DLS. All experiments were performed in triplicate.

### Transwell BBB model

To investigate BBB permeability using an *in vitro* model, Bend.3 cells were cultured in the upper compartment of a transwell device to form a tight junction (10^5^ cells/well, pore size 0.4 μm) [32, 33]. Primary astrocytes (5 × 10^4^ cells/well) were cultured in the lower compartment and incubated at 5%CO_2_ and 37°C. The successful construction of an *in vitro* BBB model was confirmed when the transendothelial electrical resistance (TEER) value exceeded 300 Ω/cm^2^ [34].

### Cellular uptake

Primary astrocytes, Bend.3 cells, and RAW264.7 cells were seeded in 24-well plates at a density of 1 × 10^5^ cells per well and incubated overnight. An equal amount of FITC‑labeled nanoformulation was added to all wells and mixed thoroughly with fresh complete medium. Additionally, a transwell BBB model was established in wells seeded with primary astrocytes, and an equal amount of FITC‑labeled nanoformulation was added to the upper chamber. After 24 h, the cells were washed three times with PBS. Sufficient cells were collected for flow cytometry analysis, while the remaining cells were imaged using a Thunder microscope. All experiments were performed in triplicate.

### *In vitro* patch-clamp recordings

Whole-cell patch-clamp recordings were performed on RAs at 0, 15, and 30 days post-R/T-MaM-NPs induction to validate neuronal excitability. The experimental workflow was as follows: cell-attached coverslips were transferred to a perfusion chamber continuously superfused (2 mL/min, 32 ± 0.5°C) with oxygenated artificial cerebrospinal fluid containing (in mM): 125 NaCl, 25 NaHCO_3_, 2.5 KCl, 1.25 NaH_2_PO_4_, 2 CaCl_2_, 1.5 MgCl_2_, and 10 d-glucose (pH 7.4; osmolality 300–310 mOsm). Borosilicate glass patch electrodes were fabricated using a Flaming-Brown puller (Sutter Instruments, CA) and filled with internal solution (in mM): 135 K-gluconate, 5 KCl, 10 HEPES, 2 EGTA, 4 ATP, 0.5 GTP (osmolality 280–290 mOsm, pH 7.4), yielding 4–7 MΩ resistance. Recordings were acquired via an Axopatch 700B amplifier (Axon Instruments, Sunnyvale, CA). Action potentials were evoked by incremental depolarizing current injections in current-clamp mode. Potassium currents were recorded in voltage-clamp mode with the membrane potential held at -70 mV. Drug delivery utilized a gravity perfusion system (VC-6, Harvard Apparatus, Hamden, USA). Data acquisition and analysis were performed through a Digidata 1440 interface (Axon Instruments) using pClamp 10.0 software (Axon Instruments).

### Experimental animals

Closed-colony ICR mice (6–8 weeks old, male) were purchased from the Animal Experiment Center of Nantong University. Before and during the experiment, the ARRIVE guidelines were followed. The animal room was kept at a constant temperature of 22°C, with a humidity of 50–60% under a standard light/dark cycle of 12 h. The mice had free access to food and water. All animal procedures were approved by the Nantong University Laboratory Animal Ethics Committee (Approval no. S20250507-002).

### Establishment of TBI models

Referring to the literature [35], controlled cortical injury was used to simulate the TBI model in mice. Briefly, after mice were induced anesthetized using 3% isoflurane, isoflurane was reduced to 1.5% to maintain anesthesia. The head of the mice was fixed on the stereotaxic apparatus with a heat pack underneath to prevent the mice from being cooled. An incision was made in the middle of the scalp to create a 2-mm-diameter window (location: 2.0-mm lateral to the median sagittal line and 1.0-mm posterior to the posterior fontanel), and the brain was impacted vertically using an impactor with the following impinging parameters: impact velocity, 3.5 m/s; deformation depth, 1.30 mm; and duration, 0.4 s. The brain was rinsed clean with physiological saline after the impact was completed, and the hemorrhage was stopped with a sterile cotton swab. The wound was then sutured and anti-infected with antibiotic ointment.

### Whole-body biodistribution and metabolism of nanomaterials

TBI model mice were divided into six groups (PBS, free‑DiR, NPs‑DiR, Man‑NPs‑DiR, R/T‑MaM‑NPs‑DiR, and R/T‑MaM‑NPs‑DiD; *n* = 6) to evaluate the *in vivo* distribution of the formulations. The respective nanoformulations (R/T‑MaM‑NPs‑DiR at a standard dose of 20 mg/kg) were administered via tail‑vein injection. Twenty‑four hours post‑injection, whole‑body distribution was monitored using IVIS fluorescence imaging. Mice were then euthanized without cardiac perfusion, and major organs (heart, liver, spleen, lung, kidney, and brain) were collected to examine metabolic profiles. For the R/T‑MaM‑NPs‑DiD group, brain tissues were processed into frozen sections, and the distribution of R/T‑MaM‑NPs within the brain was observed under an inverted Leica microscope.

### Blood circulation time of NPs

Healthy ICR mice (6–8 weeks old, male) were divided into four groups (free-DiR group, NPs-DiR group, MaM-NPs-DiR group, and R/T-MaM-NPs-DiR group; *n* = 6). An equal dose of DiR‑labeled NPs was administered via tail‑vein injection. The systemic distribution of the NPs was then monitored using an IVIS fluorescence imaging system at 1, 7, 14, 21, and 28 days post‑injection to evaluate their circulation time in the blood.

### Immunostaining and H&E staining

Cells or brain tissues were fixed in 4% paraformaldehyde for 30 min. Brain tissues were then dehydrated in 30% sucrose solution, and 12-μm sections were prepared using a cryostat. Sections were blocked with 5% serum in 0.5% Triton X‑100 for 2 h, followed by incubation with primary antibodies at 4°C overnight. After washing three times with PBS, sections were incubated with fluorescent secondary antibodies and counterstained with DAPI. Apoptosis in the injury area was detected using a TUNEL assay kit (C1089, Beyotime, China). Detailed information on antibodies used is provided in Supplementary Table S1.

For hematoxylin & eosin (H&E) staining, tissue sections were progressively hydrated through a descending alcohol series, rinsed with distilled water, stained with hematoxylin for 10–15 min, rinsed under running tap water, stained with 0.5% eosin for 2–5 min, dehydrated through an ascending alcohol series, cleared in xylene, and finally mounted with coverslips. Images were acquired using an inverted Leica microscope (DM 5000 B; Leica) and analyzed with ImageJ software. HE-stained sections were examined using ImageJ to determine the extent of TBI-related damage in the ipsilateral cortex. The percentage of tissue loss in the hemisphere was reported according to the following calculation: (contralateral hemisphere area − ipsilateral hemisphere area)/contralateral hemisphere area × 100%.

### Quantitative real‑time PCR

Thirty-two days post-nanoformulation treatment, RAs were isolated from the ipsilateral hemisphere of TBI-injured mouse brains by flow cytometry sorting. Total RNA was extracted from RAs using the FastPure Cell/Tissue Total RNA Isolation Kit V2 (Vazyme Biotech Co., Ltd., Nanjing, China). Subsequently, 1000 ng of total RNA was reverse-transcribed using a reverse transcription kit (Thermo Fisher Scientific, Waltham, MA, USA). cDNA derived from cells was diluted 10-fold, respectively, for subsequent analyses. Quantitative real-time PCR (qRT-PCR) was performed using Q5 (Thermo Fisher Scientific) with GAPDH as the internal reference for mRNA. PCR primer sequences are listed in Supplementary Table S2. Data analysis was conducted using the 2-ΔΔCT method.

### Enzyme-linked immunosorbent assay

Cytokine levels [IL-1β, IL-6, tumor necrosis factor (TNF)-α, IL-4, transforming growth factor (TGF)-β, and IL-10] in ipsilesional cerebral hemispheres were quantified using commercial enzyme-linked immunosorbent assay (ELISA) kits (Sabbiotech, USA). Absorbance was measured at 450 nm with a microplate reader (Biotek Instruments, USA), and optical density values were converted to corresponding concentration units based on standard curves.

### *In vivo* MRI

TBI mice were divided into five groups: Sham, PBS, R/T‑MaM‑NPs without Rg1, R/T‑MaM‑NPs, and Oral Rg1 (*n* = 6). Magnetic resonance imaging (MRI) was performed using a 3.0-T MRI scanner both before and 1 day after tail‑vein injection of the respective nanoformulations (R/T‑MaM‑NPs, standard dose 20 mg/kg). T2‑weighted images (T2WI) were acquired to assess cerebral edema post‑TBI. After 32 days of treatment, T1‑weighted images (T1WI) were obtained to evaluate changes in lesion size following long‑term therapy. T1WI parameters: TE 4.0 ms, TR 8.9 ms, slice gap 1.0 mm, field of view (FOV) 8 cm. T2WI parameters: TE 96.0 ms, TR 3800 ms, slice gap 1.6 mm, FOV 8 cm.

### Brain water content measurement

Mice in different groups were treated with an equal dose of nanoformulations after TBI (*n* = 6), and the extent of cerebral edema was assessed 1 day post‑treatment. The procedure was as follows: after euthanasia, brain tissue was harvested without perfusion. The wet weight was measured using a microbalance. The samples were then dried at 100°C for 48 h to obtain the dry weight. Brain water content was calculated using the following formula:


Brain water content (%)=[(Wet weight−Dry weight)/Wet weight]×100%

### Morris water maze test

The Morris water maze experiment was performed 28–32 days after TBI to test the spatial learning and memory abilities of mice. The water maze divided the pool into four quadrants, which were filled with water at about 24°C. A small amount of a non-toxic black additive was added to make the water opaque, and a 10-cm-diameter target platform was placed in the second quadrant at a height of 1 cm below the water surface. The mice were trained for four consecutive days, four times a day, half an hour apart, with each training session in each of the four quadrants as an initial point. If the mouse found the platform within 90 s, it was allowed to stay on the platform for 15 s; otherwise, the mouse was guided to the platform and allowed to stay there for 30 s. On the fifth day, the test experiment was performed. Before the test, the platform was removed, and the mouse was allowed to swim freely in the pool for 90 s. The EthoVision XT software was used to record the swimming trajectory of the mice and analyze the latency to escape in seconds and the number of times the platform position was crossed.

### Assessment of neurological deficits

The neurological deficits of TBI mice after treatment were assessed using a modified neurological severity score (mNSS). The mNSS score ranges from 0 to 18 points and is divided into three levels: 1–6 points indicate mild impairment, 7–12 points indicate moderate impairment, and 13–18 points indicate severe impairment. Each aspect is assessed separately: motor, sensory, reflex, and balance functions. Moreover, neurological function was independently assessed by two researchers who were not informed about the treatment allocation.

### Rotarod test

Mice were tested in the rod-turning test 1 day before TBI and 1, 7, 14, and 28 days after TBI. Before the test, the mice were trained on a rotating rod at an accelerated speed (5–30 rpm) for 3 days. The mice were trained five times a day for 300 s each time, with an interval of 5 min, and the latency of each fall of the mouse from the rotating rod was recorded. During the testing phase, the mice were directly tested on a rotating rod at a constant speed (30 rpm), and the latency of each mouse to fall was recorded, with a maximum time of 300 s. All experiments and data analyses were performed using a blind method.

### *In vivo* biological safety assessment

Healthy 8-week-old ICR mice (*n* = 6) were selected. Rg1, PBS, R/T-MaM-NPs, and R/T-MaM-NPs (without Rg1) (R/T-MaM-NPs, standard dose 20 mg/kg) were injected into the tail vein of the mice. Samples of the main organs of mice were collected 7 days after injection. After staining the organs with H&E, the tissue structure and cell morphology were observed under a microscope to assess the potential toxicity and adverse effects of nanomaterials on mice. All safety assessment indicators used the single-blind method.

### Statistical analysis

Statistical analyses and graphical representations were performed using GraphPad Prism 10.1.2. Data are presented as mean ± standard deviation (SD). Intergroup comparisons were conducted using unpaired two-tailed Student’s *t*-tests; multigroup analyses employed one-way analysis of variance (ANOVA) with Tukey’s *post hoc* test for multiple comparisons. All longitudinal behavioral data were analyzed by two-way ANOVA followed by Tukey’s *post hoc* test. All experiments were repeated at least thrice.

## Results and discussion

### Preparation and characterization of R/T-MaM-NPs nanosystem

**Figure 2:**
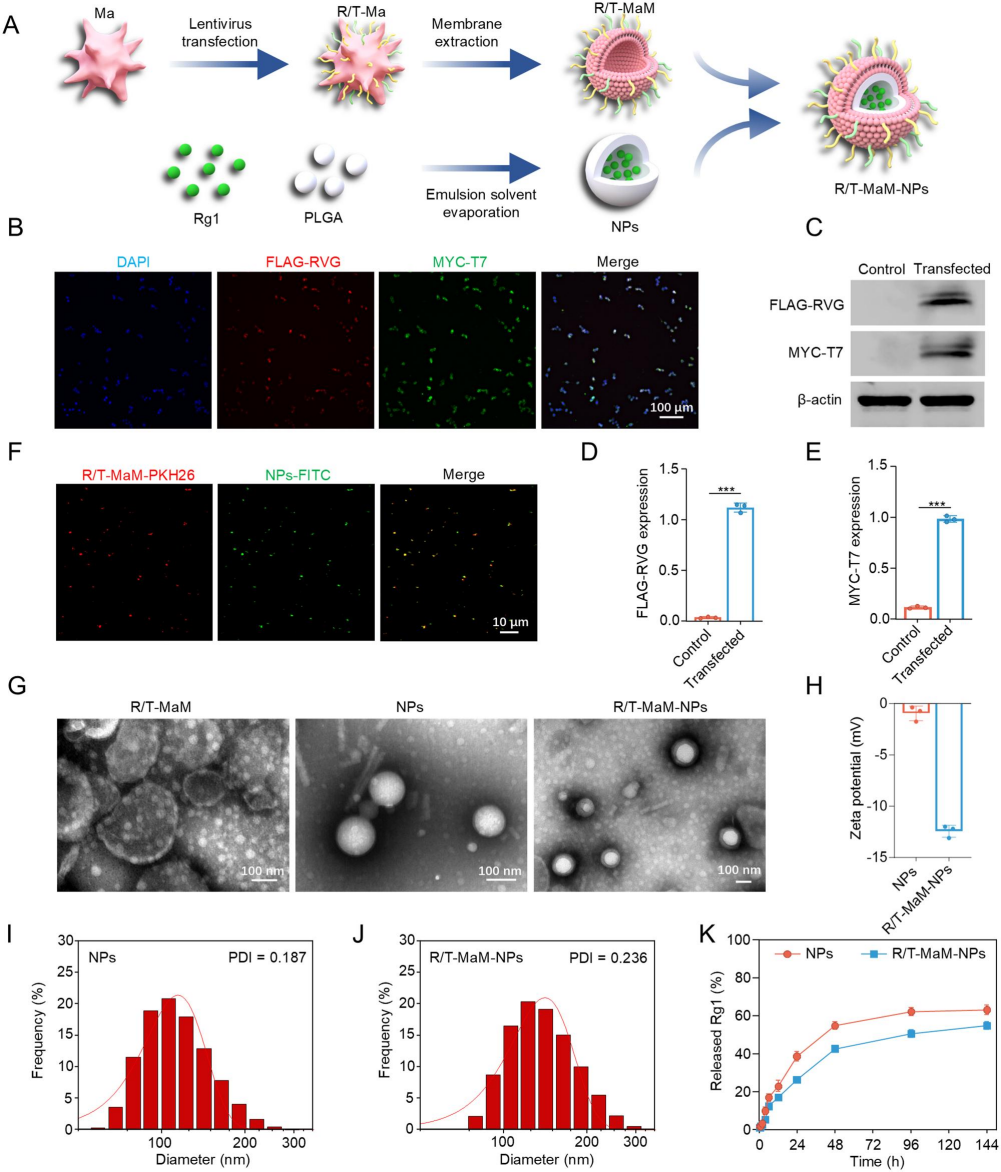
Synthesis and characterization of the nanotherapeutic. (**A**) Schematic of RAW264.7 macrophage (Ma) transfection with RVG-FLAG/T7-MYC-expressing lentivirus, membrane extraction (R/T-MaM), and nanoformulation preparation. (**B**) Representative immunofluorescence images of engineered RAW264.7 cells co-expressing FLAG and MYC tags; nuclei counterstained with DAPI. Scale bar: 100 μm. (**C**) Western blot (WB) validation of FLAG/MYC expression in R/T-MaM. (**D and E**) Quantitative analysis of data in (**C**). (**F**) Fluorescent overlay showing PKH26-labeled R/T-MaM encapsulating FITC- labeled NPs. Scale bar: 10 μm. (**G**) Transmission electron microscopy (TEM) images of R/T-MaM, NPs, and R/T-MaM-NPs. Scale bar: 100 nm. (**H**) Zeta potential of NPs and R/T-MaM-NPs. (**I and J**) Hydrodynamic diameter distribution of NPs and R/T-MaM-NPs. (**K**) Rg1 release kinetics in PBS (pH 7.4, 37°C). Data are presented as mean ± SD (**D, E, H, and K**) *n* = 3 (***P < 0.001).

Fabrication of brain-targeted biomimetic nanosystem: an efficient strategy for overexpression of targeting peptides on cell membranes via genetic engineering (Figure 2A). As shown in Supplementary Figure S1A, we redesigned the gene sequence to fuse the transmembrane domain at the C terminus of RVG and T7 dual peptides. This modification anchors proteins to the cell membrane, serving as a bridge between extracellular and intracellular compartments to ensure proper membrane insertion. RVG functions as the targeting moiety by binding to the AChRs, which are extensively expressed on neural cells in the brain, including astrocytes. The T7 peptide exhibits high binding affinity with the TfRs on the BBB. Furthermore, FLAG and MYC epitope tags were incorporated at the C-terminal ends of RVG and T7 peptides, respectively, facilitating subsequent validation of peptide expression. Lentiviral transduction, which enables stable overexpression of target peptides through cellular infection, represents the optimal strategy for acquiring sufficient cell membranes required for fabricating this biomimetic nanosystem [36]. Flow cytometric analysis revealed approximately 82.72% transduction efficiency of control lentivirus in RAW264.7 cells at 72 h post-transduction (Supplementary Figure S1B–D). Following 72-h infection of RAW264.7 cells with genetically engineered lentivirus, immunofluorescence staining demonstrated that the majority of cells co-expressed FLAG and MYC tags (Figure 2B). This demonstrates successful co-expression of RVG and T7 dual peptides. Western blot (WB) analysis further confirmed significant upregulation of RVG-FLAG and T7-MYC expression in transfected groups compared to controls (Figure 2C–E). Collectively, these findings validate the successful generation of RVG/T7-expressing RAW264.7 macrophages (designated as R/T-Ma). Next, R/T-Ma cells were polarized toward M2 phenotype using 10 ng/mL IL-4. Flow cytometry confirmed expression of M2 macrophage markers CD206 or arginase-1 (Arg-1) in polarized cells (Supplementary Figure S1E).

Subsequently, we employed PLGA to fabricate biocompatible nanocarriers with high DL capacity for Rg1 encapsulation [37]. Rg1 is an herbal constituent with neuroprotective properties and astrocyte transdifferentiation-promoting effects. The synthesized NPs exhibited spherical morphology under electron microscopy (Figure 2G and Supplementary Figure S1F), with a mean diameter of 116.6 ± 34.0 nm and zeta potential of −0.72 ± 5.44 mV (Figure 2H and I). Quantitative analysis based on the Rg1 standard curve (Supplementary Figure S1G) demonstrated an EE of 74.3% and DL capacity of 15.6%. Following R/T-MaM coating, the NPs maintained spherical core-shell structures (Figure 2G and Supplementary Figure S1F), showing increased diameter (141.9 ± 40.5 nm) and altered zeta potential (−12.43 ± 13.50 mV) as depicted in Figure 2H and J. The 25-nm diameter increment was attributed to the R/T-MaM membrane coating (consistent with the size change of NPs after labeling with fluorescent dye, Supplementary Figure S2A and Supplementary Table S3). Furthermore, to determine the MaM coating efficiency of the NPs, NPs and R/T-MaM were labeled with FITC (green) and PKH26 (red) fluorescent dyes, respectively. R/T-MaM-NPs prepared using different formulation ratios were examined under fluorescence microscopy, which confirmed successful coating of the NPs by the R/T-MaM membrane. The highest coating efficiency (approximately 56%) was achieved at a 1:2 ratio (NPs:R/T-MaM) (Figure 2F, Supplementary Figure S2B and C), and this formulation was used in subsequent experiments. Due to the relatively small size difference before and after coating, efficient separation and purification of the coated NPs using conventional methods remains challenging, representing an area for improvement in future studies.

To assess membrane protein integrity post-coating, SDS-PAGE analysis was performed to compare protein profiles before and after membrane encapsulation. The results revealed that R/T-MaM-NPs retained all characteristic protein bands of native MaM (Supplementary Figure S2D), confirming not only successful membrane coating but also preservation of native membrane protein composition. Furthermore, the biomimetic membrane coating endows R/T-MaM-NPs with favorable plasma stability and a sustained-release drug profile (Supplementary Figure S2E and F, Figure 2K), effectively reducing drug leakage during transport.

To investigate the drug release mechanism, the Rg1 release profiles were fitted with multiple mathematical models, including zero-order, first-order, Higuchi, and Ritger–Peppas models (Table 1). The release kinetics analysis indicated that all systems followed first-order kinetics (*R*^2^ > 0.995), suggesting that the release process was predominantly diffusion-controlled. Further analysis using the Ritger–Peppas model revealed that the release from uncoated NPs approximated Fickian diffusion (*n* ≈ 0.445), whereas the *n* value for membrane-coated NPs (R/T-MaM-NPs) increased to approximately 0.5. This shift indicates a transition to non-Fickian diffusion, implying that the coating not only acts as a diffusion barrier to retard drug release but may also enhance release controllability and sustainability through dynamic interfacial interactions of the membrane or synergistic polymer–coating modulation, thereby achieving improved sustained-release performance.

**Table 1:** Release models for NPs and R/T-MaM-NPs.

	NPs	R/T-MaM-NPs
Zero order	*y* = 12.52 + 0.45*x* *R* ^2^ = 0.743	*y* = 8.34 + 0.39*x* *R* ^2^ = 0.822
First order	*y* = 63.69(1−e^−0.041^^*t*^) *R* ^2^ = 0.997	*y* = 54.98(1−e^−0.03^^*t*^) *R* ^2^ = 0.995
Higuchi	*y* = 6.2*t*^0.5^ + 0.07 *R* ^2^ = 0.920	*y* = 5.28*t*^10.5^ − 1.94 *R* ^2^ = 0.960
Ritger–Peppas	*y* = 7.84*t^n^* *R* ^2^ = 0.929 *n* = 0.445 ± 0.058	*y* = 5.04*t^n^* *R* ^2^ = 0.956 *n* = 0.499 ± 0.052

Collectively, we successfully synthesized a brain-targeting, stable, and sustained-release biomimetic nanosystem (R/T-MaM-NPs).

### *In vitro* cellular uptake of R/T-MaM-NPs nanosystem

**Figure 3:**
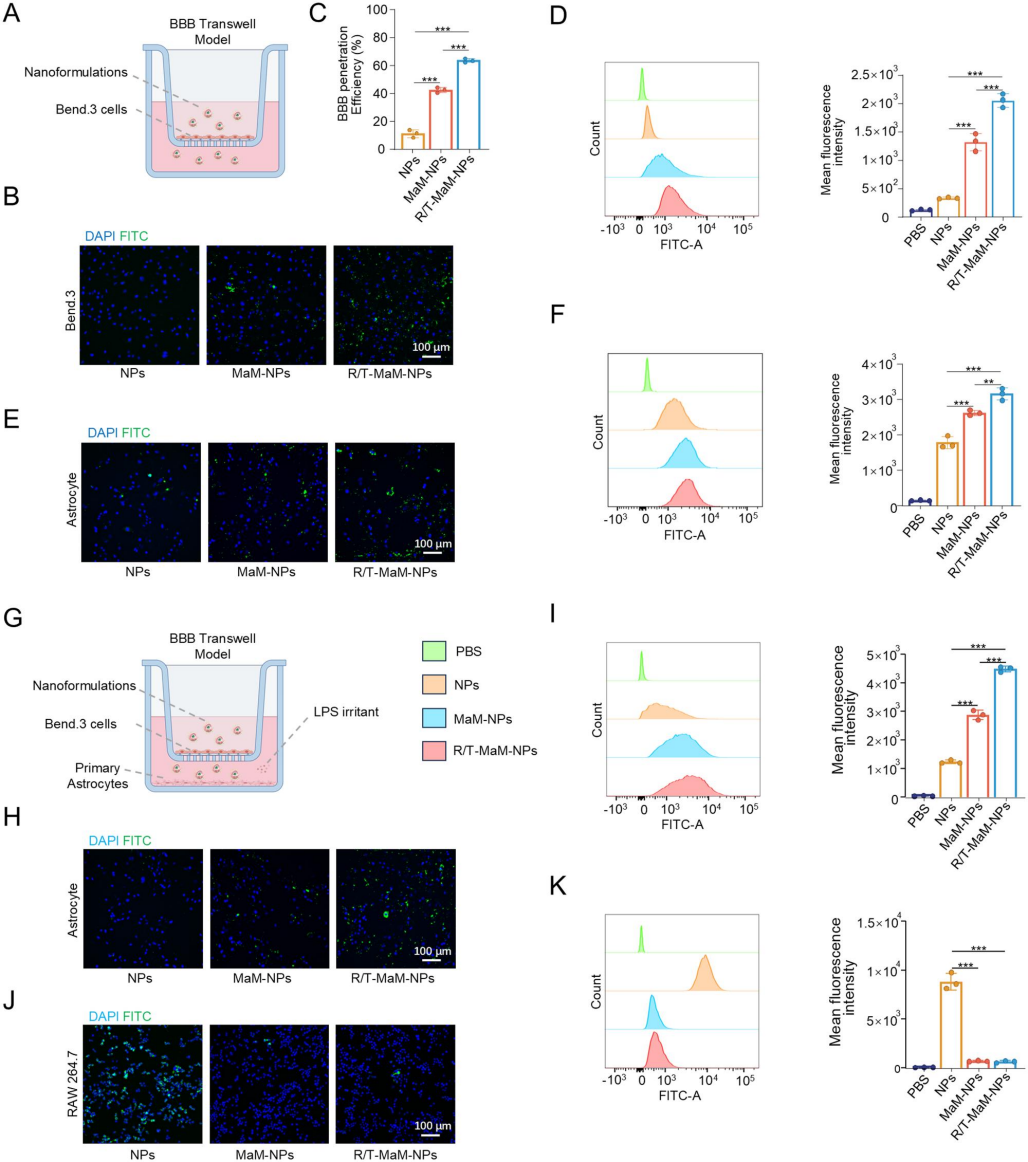
*In vitro* cellular uptake of nanoformulations. (**A**) Schematic of the *in vitro* BBB transwell model by figdraw.com. (**B**) Representative fluorescence images of FITC-labeled nanotherapeutics internalized by Bend.3 cells (brain endothelial cells) and (**D**) corresponding quantitative analysis by flow cytometry. Nuclei are stained with DAPI. Scale bar: 100 μm. (**C**) Analysis of BBB penetration efficiency for different nanoformulations. (**E**) Representative fluorescence images of FITC-labeled nanotherapeutics internalized by primary astrocytes and (**F**) corresponding quantitative analysis by flow cytometry. Nuclei are stained with DAPI. Scale bar: 100 μm. (**G**) Schematic of the *in vitro* BBB transwell model simulating post-TBI astrocytic uptake. (**H**) Representative immunofluorescence images showing the internalization of FITC-labeled nanotherapeutics by primary astrocytes in the lower chamber under the setup in (**G**) and (**I**) corresponding quantitative analysis by flow cytometry. Nuclei are stained with DAPI. Scale bar: 100 μm. (**J**) Representative fluorescence images of the immune evasion co-culture assay with FITC-labeled nanoformulations and (**K**) corresponding quantitative analysis by flow cytometry. Nuclei are stained with DAPI. Scale bar: 100 μm. Data are presented as mean ± SD; (**C, D, F, I, and K**) *n* = 3 (**P < 0.01, ***P < 0.001).

The tight junctions between vascular endothelial cells within the BBB constitute the primary biological obstacle for drug delivery [38]. To investigate how biomimetic nanoformulations cross this barrier, we established a transwell BBB model by culturing Bend.3 cells in the upper chambers (Figure 3A). The integrity of Bend.3 cell layers was confirmed by achieving TEER values ≥300 Ω/cm^2^ [39]. Fluorescent labeling of biomimetic NPs with FITC was implemented to visualize both BBB penetration efficiency and cellular uptake dynamics.

To validate the high-affinity interaction between T7 peptide and TfR on brain microvascular endothelial cells, co-culture experiments with Bend.3 cells revealed that R/T-MaM-NPs exhibited markedly higher fluorescence intensity than both MaM-NPs and bare NPs groups (with the latter showing minimal signal) (Figure 3B and D). To exclude potential interference from the RVG peptide, single-peptide control groups (T7-MaM-NPs and RVG-MaM-NPs) were compared with R/T-MaM-NPs. Results show that for RVG-MaM-NPs modified solely with RVG peptide, T7 peptide-mediated BBB targeting promotes Bend.3 cellular uptake of T7-MaM-NPs and R/T-MaM-NPs, and RVG peptide minimally affects the BBB targeting function of T7 peptide (Supplementary Figure S3A, C and E). This outcome demonstrates that T7 peptide effectively directs BBB-specific targeting and promotes efficient cellular uptake of the biomimetic nanoformulations. To evaluate the penetration efficiency of nanoformulations across the BBB, equal amounts of FITC‑labeled nanoformulations were added to the upper chamber of a transwell BBB model. After 24 h, the medium in the lower chamber was collected, and its fluorescence intensity was quantified. The results showed that, compared with the plain NPs group, R/T‑MaM‑NPs exhibited the highest BBB penetration rate (approximately 63%), whereas NPs lacking both the biomimetic membrane coating and dual‑peptide modification showed only 11% penetration (Figure 3C). These findings strongly demonstrate that T7‑peptide modification effectively promotes the targeting and penetration of R/T‑MaM‑NPs across the BBB.

Next, to verify the high-affinity interaction between the RVG peptide and AChRs on astrocytes, equal amounts of FITC‑labeled nanoformulations were co‑cultured with primary astrocytes. Cellular uptake of NPs was quantified via fluorescence imaging and flow cytometry. The results showed that, compared with the NPs group and the MaM‑NPs group, the R/T‑MaM‑NPs group with RVG‑peptide modification exhibited the highest fluorescence intensity, while the NPs group showed the lowest intensity (Figure 3E and F). These results indicate that RVG‑peptide modification enhances the internalization of R/T‑MaM‑NPs by astrocytes.

After investigating the *in vitro* BBB penetration and dual‑peptide targeting capability of R/T‑MaM‑NPs, we further evaluated their ability to target astrocytes cultured in the lower chamber of the transwell BBB model following barrier penetration. Low-dose LPS (1 μg/mL) was added to the lower chamber to mimic post-TBI inflammatory microenvironment, with equivalent doses of nanoformulations administered in the upper chamber. Immunofluorescence and flow cytometry analyses demonstrated significantly enhanced uptake of R/T-MaM-NPs in primary astrocytes compared to both NPs and MaM-NPs groups. Notably, MaM-NPs groups exhibited substantially higher cellular internalization than NPs groups (Figure 3G–I). Furthermore, comparative analysis between single-peptide control RVG-MaM-NPs and R/T-MaM-NPs revealed significantly reduced uptake by primary astrocytes in the lower chamber due to the absence of T7 peptide-mediated BBB targeting **(**Supplementary Figure S3B, D and F). These findings confirm that RVG/T7 dual peptides not only facilitate targeted astrocyte delivery but also markedly enhance NP uptake efficiency. Moreover, the MaM coating synergistically improves BBB permeation through its intrinsic biocompatibility and inflammatory chemotaxis properties toward lesion sites. Despite the preliminary *in vitro* validation of the dual‑peptide functionality, when translated to *in vivo* application, the simultaneous presentation of both ligands on the same membrane interface may still raise concerns regarding potential spatial steric hindrance or competitive density that could interfere with their binding efficiency to the respective receptors.

Additionally, to simulate the anti-phagocytic potential of nanoformulations during circulatory transport, we conducted co-culture experiments with RAW264.7 macrophages using distinct NP formulations. The results demonstrated significantly reduced macrophage sequestration of R/T-MaM-NPs and MaM-NPs compared to NPs, attributable to the biomimetic camouflage effect of MaMs **(**Figure 3J and K**)**. This indicates that MaM coating substantially enhances immune evasion capability, thereby minimizing NP clearance during circulatory transport.

Furthermore, the biocompatibility of nanoformulations was evaluated using the standardized CCK-8 assay. The results demonstrated negligible cytotoxicity toward primary astrocytes, Bend.3, Bv2 microglia, and Ht22 neuronal cells after 36 h co-incubation with equivalent nanoformulation concentrations (R/T-MaM-NPs at a standard dose of 300 μg/mL) (Supplementary Figure S3G).

### *In vivo* distribution of R/T-MaM-NPs nanosystem and accumulation at sites of brain injuries

**Figure 4:**
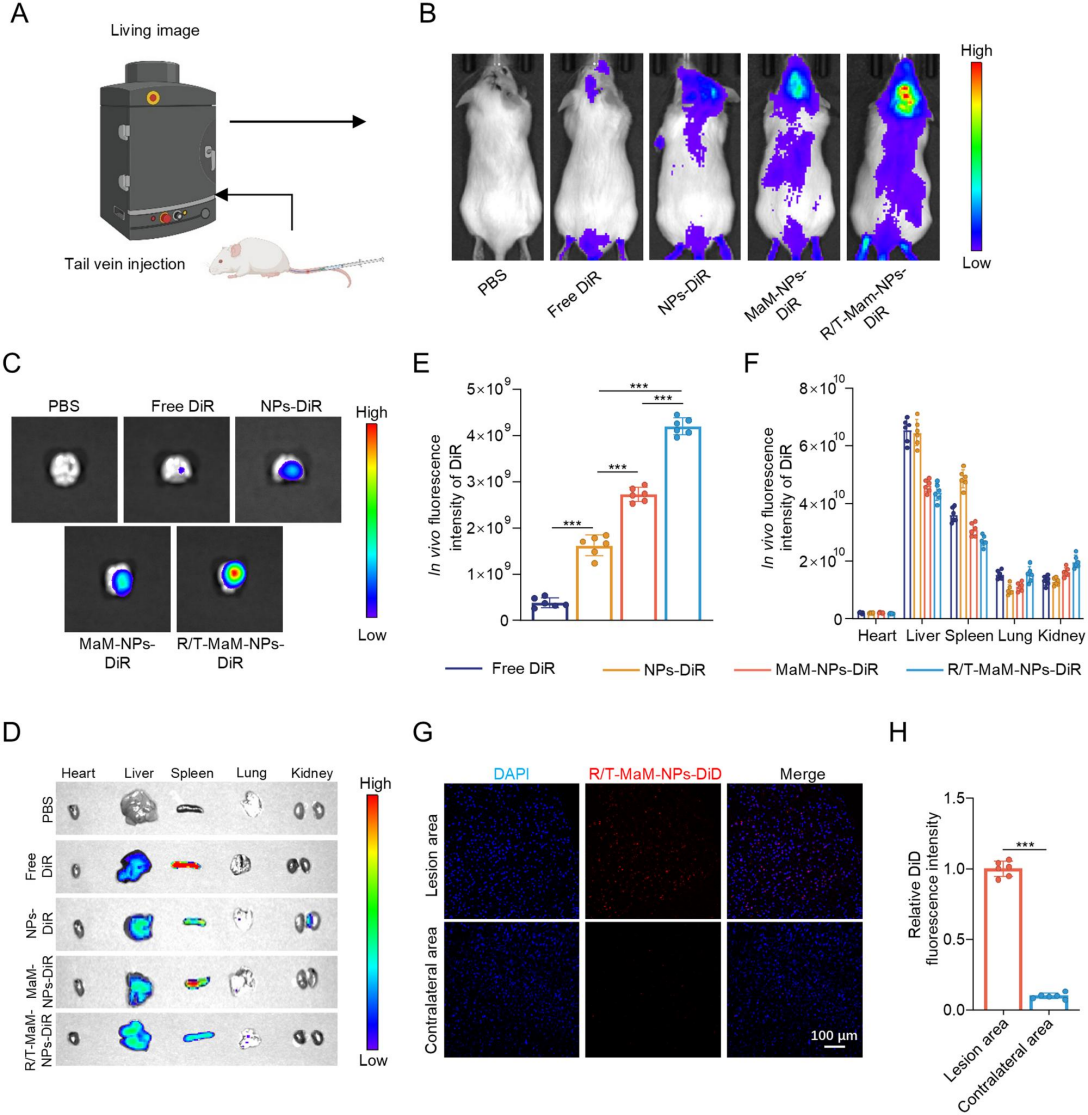
*In vivo* brain‑targeted delivery of nanoformulations. (**A**) Experimental design for *in vivo* tracking of DiR‑labeled nanoformulations. “Created in BioRender. Liao, W. (2026) https://BioRender.com/l55lq26.” (**B**) Whole‑body biodistribution profiles of TBI mice 24 h post‑injection with DiR‑labeled nanoformulations, acquired via IVIS imaging, alongside representative fluorescence images of (**C**) isolated brain tissues and (**D**) major organs. (**E and F**) Quantitative analysis of DiR signal intensity in brain (**C**) and organs (**D**). (**G**) Representative fluorescence images of the ipsilateral lesion versus the contralateral hemisphere 24 h after intravenous injection of R/T‑MaM‑NPs‑DiD, Scale bar: 100 μm, and (**H**) corresponding quantitative analysis of fluorescence intensity. Data represent mean ± SD; (**E, F, and H**) *n* = 6 (***P < 0.001).

To evaluate cerebral targeting efficacy *in vivo*, DiR-labeled nanoformulations (PBS, free DiR, NPs, MaM-NPs, and R/T-MaM-NPs) were intravenously administered via the tail vein to TBI model mice. *In vivo* imaging revealed significantly enhanced fluorescence intensity in the brains of R/T-MaM-NPs-treated mice compared to NPs and MaM-NPs groups at 24 h post-injection (Figure 4A and B). For precise quantification of cerebral DiR accumulation, brain tissues were harvested for *ex vivo* imaging and fluorescence intensity analysis across experimental groups. *Ex vivo* brain tissue imaging revealed that R/T-MaM-NPs displayed the highest DiR fluorescence signal in brain tissue (approximately 2.6-fold that of the NPs group) and preferentially localized to the TBI lesion area (Figure 4C and E). Quantitative analysis indicated that about 7% of the intravenously administered R/T-MaM-NPs reached the cerebral lesion site (Supplementary Figure S4A and B). Owing to the inflammatory tropism of MaM, the MaM-NPs group demonstrated substantially higher fluorescence intensity compared to bare NPs. *Ex vivo* imaging of major organs (heart, liver, spleen, lungs, and kidneys) revealed predominant NP accumulation in hepatic and splenic tissues (Figure 4D and F). This distribution pattern indicates that hepatic metabolism and renal clearance constitute primary elimination pathways for the nanoformulations. Thirty days after tail vein injection, the mice were imaged again *in vivo*, and the results showed that the accumulation of DiR in the brain in the R/T-MaM-NPs group was still much greater than that in the other groups, although the overall fluorescence intensity was weakened (Supplementary Figure S4C–E).

To directly visualize the distribution of R/T‑MaM‑NPs in brain tissue, mice were intravenously injected with DiD‑labeled R/T‑MaM‑NPs after TBI. 24 h later, the mice were perfused, brains were harvested, and frozen sections were prepared. Fluorescence images clearly revealed strong DiD red fluorescence signals highly concentrated in the perilesional area of the ipsilateral (right) hemisphere. In contrast, only weak nonspecific background fluorescence was observed in the contralateral (left) uninjured hemisphere, forming a sharp contrast with the signals in the lesion region (Figure 4G and H). These results provide compelling evidence for the inflammatory targeting of R/T‑MaM‑NPs to the cerebral lesion.

We hypothesized that the prolonged accumulation of R/T-MaM-NPs at the brain lesion site *in vivo* is attributable to the camouflage effect of the cell membrane coating. Healthy ICR mice were divided into four groups (free-DiR group, NPs-DiR group, Man-NPs-DiR group, and R/T-MaM-NPs-DiR group). An equal dose of DiR-labeled NPs was injected into the tail vein, and then the distribution of NPs in the whole body of the mice was observed by IVIS fluorescence imaging at 1, 7, 14, 21, and 28 days after injection. The results showed that the DiR fluorescence signal was strongly manifested in the whole body of the mice by time-dependent whole-body imaging. This indicates that all three nanomaterials achieved efficient systemic cycling. Analysis of biodistribution and retention showed that, in contrast to the rapidly cleared free DiR and conventional NPs, the DiR signals carried by MaM-encapsulated Man-NPs-DiR and R/T-MaM-NPs-DiR exhibited significantly prolonged persistence within the circulatory system (Supplementary Figure S4F). This direct evidence confirms that MaM-encapsulated NPs can achieve prolonged persistence within the circulatory system, highlighting their potential for sustained drug delivery applications. The extended blood retention time is primarily attributed to the biocompatibility and low immunogenicity of the biomimetic (macrophage) membrane, which is consistent with the results obtained from *in vitro* immune evasion co-culture experiments.

These comprehensive findings demonstrate that the combined action of RVG/T7 dual peptides and MaM enables R/T-MaM-NPs to achieve BBB penetration and localized accumulation in post-TBI cerebral injury regions sustainably. This integrated strategy establishes a robust targeting nanocarrier system for the precise delivery of Rg1 to brain lesions.

### R/T-MaM-NPs facilitate the transdifferentiation of RAs into NLCs *in vitro*

**Figure 5:**
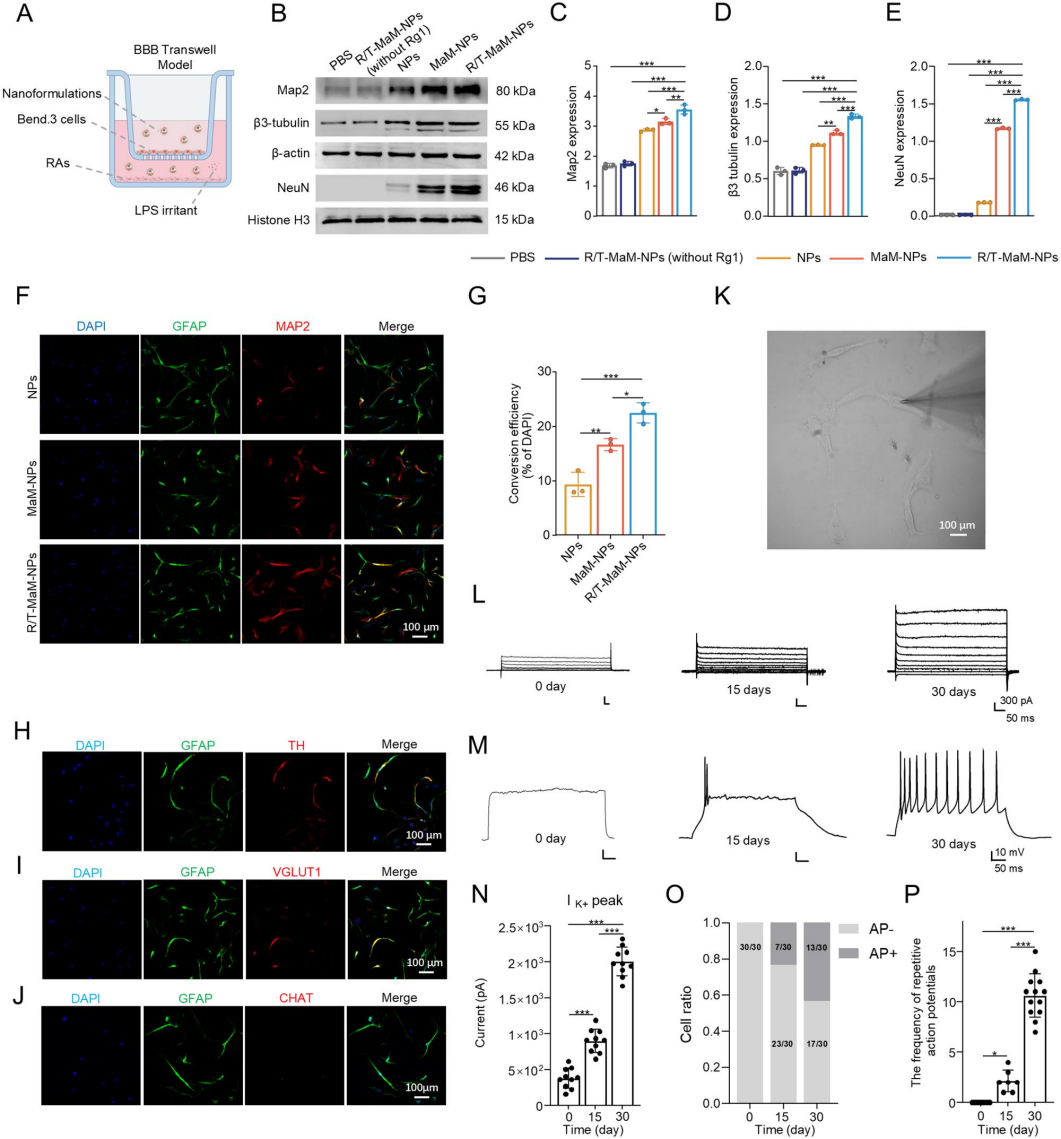
R/T-MaM-NPs Induce RAs transdifferentiation *in vitro*. (**A**) Schematic of the *in vitro* BBB transwell model. (**B**) WB analysis and (**F**) representative immunofluorescence images of neuronal markers in RAs co-cultured with nanoformulations for 7 days in a BBB transwell model. Scale bar: 100 μm. (**C–E**) Quantification of Map2, β3-tubulin, and NeuN protein levels from (**B**). (**G**) Quantitative statistics of the proportion of cells expressing Map2 for different groups of RAs in (**F**). (**H–J**) representative immunofluorescence images of RAs expressing TH, VGLUT1, and CHAT post-induction. Scale bar: 100 μm. (**K**) Field of view for whole-cell recording. Scale bar: 100 μm. (**L**) Representative potassium current traces (*I*_K+_) in transdifferentiated NLCs. Quantitative analysis shows increased peak *I*_K+_ during development (**N**). (**M**) Whole-cell recordings showing increased action potential firing in NLCs from day 0–30 after R/T-MaM-NPs induction. Quantitative results present the proportion of NLCs with repetitive firing (**O**) and firing frequency (**P**) at different time points. Data represent mean ± SD; (**C–E and G**) *n* = 3, (**N**) *n* = 10, (**O and P**) 7 < *n* < 30 (*P < 0.05, **P < 0.01, ***P < 0.001).

Previous studies have demonstrated that Rg1 can induce RAs to acquire neuronal characteristics (termed NLCs herein) [14], promoting CNS repair. However, the BBB significantly compromises its therapeutic efficacy. To explore whether the nanoformulation could cross the BBB and induce RAs transdifferentiation into NLCs after TBI in mice, we performed this *in vitro* with the aid of the transwell BBB model described above. Primary astrocytes were seeded in the lower chamber and activated into RAs via TGF-β1 stimulation [40], with LPS supplementation to mimic the inflammatory microenvironment of post-TBI lesion sites [41]. NPs, MaM-NPs, and R/T-MaM-NPs (R/T-MaM-NPs at a standard dose of 300 μg/mL) were introduced into the upper chamber to penetrate Bend.3 cells, establishing co-culture conditions with lower chamber RAs (Figure 5A). After a 7-day intervention, WB analysis of RAs lysates revealed upregulated expression of mature neuronal markers (Map2, β3-tubulin, NeuN) in the R/T-MaM-NPs group. The NPs group exhibited the lowest expression levels (Figure 5B–E). Immunofluorescence validation confirmed consistent results with WB analysis. R/T-MaM-NPs-treated RAs exhibited the highest fluorescence intensity for mature neuronal markers MAP2, βIII-tubulin, and NeuN, achieving a maximum transdifferentiation rate of 24.12%. In contrast, NPs-treated groups showed negligible expression, while MaM-NPs groups demonstrated significantly higher marker expression than NPs groups in both WB and immunofluorescence assays (Figure 5F and G, Supplementary Figure S5). Furthermore, to determine the neuronal subtypes of transdifferentiated RAs, immunofluorescence analysis revealed that Rg1-induced RAs exhibited significantly higher expression of Th and Vglut1, with virtually undetectable Chat levels (Figure 5H–J). These phenotypic markers indicate that the induced RAs primarily differentiate into dopaminergic and glutamatergic neuronal subtypes.

To provide more evidence that the induction of R/T-MaM-NPs enables transdifferentiated RAs to function as neurons, whole-cell patch-clamp recordings were performed. Electrophysiological analysis confirmed that NLCs exhibited significant potassium currents and repetitive action potential firing capabilities (Figure 5K–M). As anticipated, when NLCs cultured between 0 and 30 days after induction were analyzed, transdifferentiated NLCs showed a clear developmental time course of functional maturation: Potassium current amplitudes increased from 0.9 nA at 15 days to 2.1 nA at 30 days post-induction, with kinetic transitions from slow to fast activation implicating development of neuronal *Kv* currents (Figure 5L and N). Moreover, the proportion of cells capable of generating action potentials significantly increased over time (Figure 5O), with the frequency of repetitive firing events rising from approximately 2 events at day 15–10 events at day 30 (Figure 5P).

Based on these findings, we conclude that R/T-MaM-NPs effectively traverse the BBB, target RAs, and induce their transdifferentiation into electrophysiologically functional NLCs. This therapeutic outcome is attributed to the synergistic targeting effect conferred by the RVG/T7 dual-peptide modification coupled with the inherent chemotactic property of the MaM, which collectively enhances the uptake of R/T-MaM-NPs by RAs and promotes their subsequent transdifferentiation. Conversely, unmodified NPs are largely excluded by the BBB.

### R/T-MaM-NPs inhibit Wnt/Notch signaling to induce transdifferentiation of RAs into NLCs

**Figure 6:**
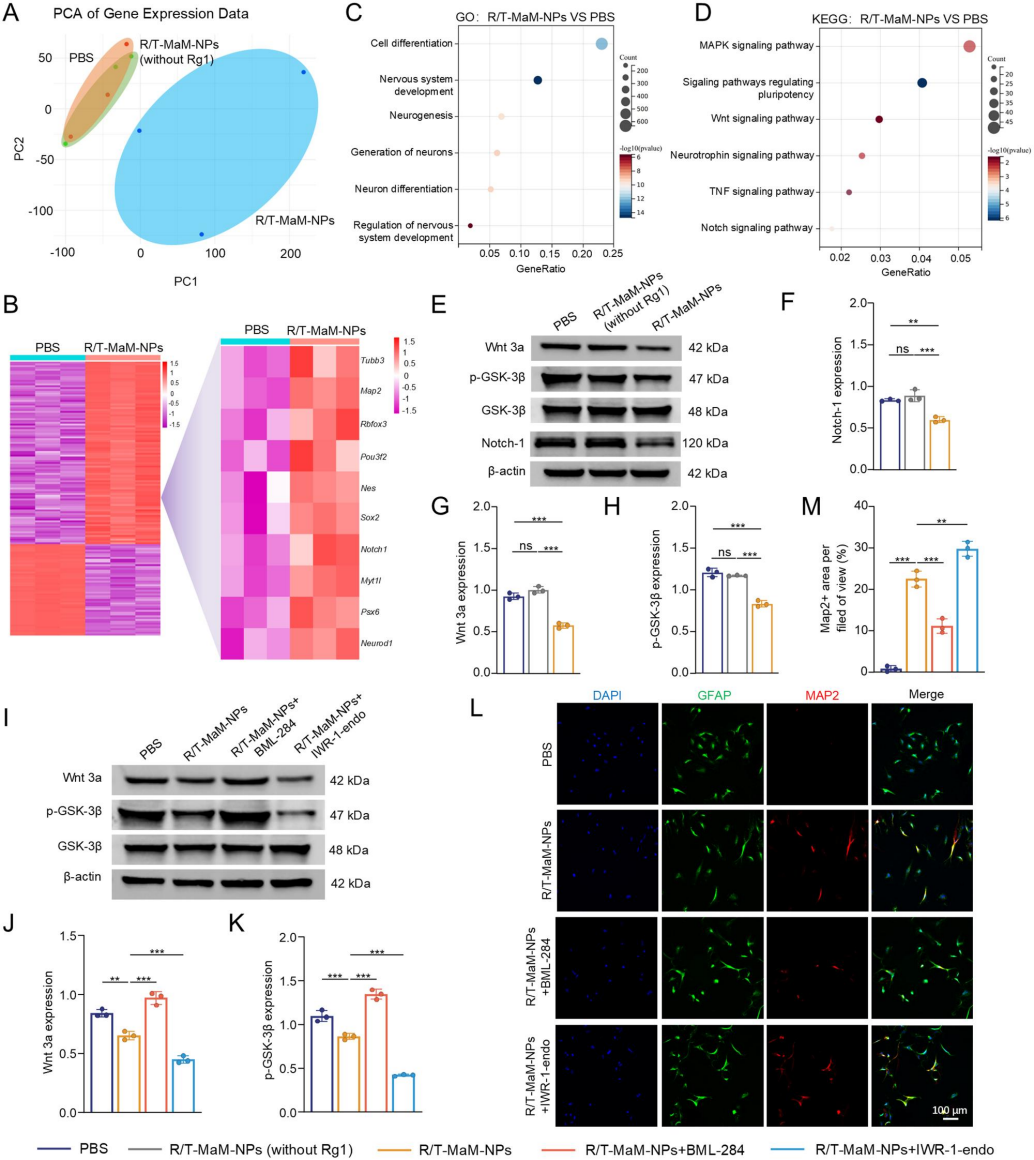
Molecular mechanisms underlying R/T-MaM-NPs-induced transdifferentiation of RAs. (**A**) Principal component analysis (PCA) of RNA-seq data from PBS, R/T-MaM-NPs (without Rg1) and R/T-MaM-NPs-treated RAs. (**B**) The heatmap displays the overall distribution of differentially expressed genes (DEGs) and associated DEGs from RNA sequencing. (**C**) Gene ontology (GO) analysis of upregulated genes in R/T-MaM-NPs-treated RAs versus PBS controls. (**D**) Kyoto Encyclopedia of Genes and Genomes (KEGG) analysis of upregulated genes in R/T-MaM-NPs-treated RAs. (**E**) WB showing expression patterns of Wnt and Notch signaling-related proteins in RAs after 7-day R/T-MaM-NPs induction, with quantitative analysis (**F–H**). (**I**) WB showing expression of associated proteins in RAs after treatment with Wnt signaling agonist BML-284 or inhibitor IWR-1-endo, with quantification (**J and K**). (**L**) Representative immunofluorescence images demonstrating Map2 expression in RAs treated with BML-284 agonist or IWR-1-endo inhibitor, with quantification (**M**). Scale bar: 100 μm. Data represent mean ± SD; (**F, G, H, J, K, and M**) *n* = 3 (**P < 0.01, ***P < 0.001, ns: not significant).

To further investigate gene expression differences in NLCs post-transdifferentiation, RNA sequencing was performed on both pre- and post-induced RAs. Principal component analysis (PCA) demonstrated spatial segregation among R/T-MaM-NPs, PBS control, and empty vector groups, indicating significant divergence in transcriptional profiles (Figure 6A). Heat map analysis showed that the upregulated genes included many genes related to early and mature neurons, such as *Tubb3*, *Map2*, *Rbfox3*, *Pou3f2*, *Nes*, and *Sox2* (Figure 6B). Gene Ontology (GO) analysis of enriched biological processes revealed that upregulated genes in R/T-MaM-NPs-treated RAs were positively associated with cellular differentiation, nervous system development, neurogenesis, neuron generation, and neuronal differentiation (Figure 6C and Supplementary Figure S6A). Kyoto Encyclopedia of Genes and Genomes (KEGG) enrichment analysis indicated that differentially expressed genes in R/T-MaM-NPs-treated RAs were enriched in pathways governing cell differentiation and proliferation, including the MAPK signaling pathway, pluripotency regulation signaling, Wnt signaling pathway, neurotrophin signaling pathway, TNF signaling pathway, and Notch signaling pathway (Figure 6D). Gene set enrichment analysis revealed significantly attenuated activity of Wnt and Notch signaling pathways in R/T-MaM-NPs-treated RAs (Supplementary Figure S6B and C).

Many studies confirm that astrocyte-to-neuron transdifferentiation is critically regulated by Wnt and Notch signaling pathways, both playing pivotal roles in cellular transformation, proliferation, and apoptosis [42–45]. WB analysis revealed significantly reduced expression of Wnt3a, p-GSK-3β, and Notch-1 in R/T-MaM-NPs-treated RAs compared to PBS controls (Figure 6E–H), suggesting that R/T-MaM-NPs promote RAs transdifferentiation by suppressing Wnt/Notch signaling. To validate this mechanism, co-treatment with Wnt agonist BML-284 and inhibitor IWR-1-endo during R/T-MaM-NPs induction was performed. BML-284 significantly increased Wnt3a and p-GSK-3β expression versus R/T-MaM-NPs alone, while immunofluorescence demonstrated BML-284 abolished transdifferentiation, markedly reducing neuronal marker Map2 expression. Conversely, IWR-1-endo substantially suppressed Wnt3a and p-GSK-3β expression, and immunofluorescence confirmed enhanced Map2 expression, potentiating transdifferentiation efficacy (Figure 6I–M).

Collectively, these findings provide conclusive evidence supporting our hypothesis that R/T-MaM-NPs suppress Wnt/Notch signaling pathways to promote transdifferentiation of RAs into NLCs.

### R/T-MaM-NPs promote astrocyte transdifferentiation in the cerebral lesion of TBI mice

Post-TBI neural pathology is predominantly characterized by neuronal apoptosis and loss, and neurons in the CNS cannot regenerate themselves after injury. Thus, the reactivation and recruitment of neurons at the site of injury constitutes a key therapeutic goal. Should R/T-MaM-NPs effectively augment RAs’ transdifferentiation in TBI-induced cerebral lesions, this would hold substantial therapeutic implications for CNS repair. We subsequently assessed the transdifferentiation potential of R/T-MaM-NPs on RAs at post-TBI cerebral lesion sites in mice. We divided mice into PBS group, empty vector group (R/T-MaM-NPs without Rg1), R/T-MaM-NPs group, and oral Rg1 group. Since inflammation at the site of brain injury generally peaks between 1 and 3 days after TBI in mice [46], we chose to perform tail vein injection of the nanoformulation (R/T-MaM-NPs at a standard dose of 20 mg/kg) 3 days after TBI in mice. This timing optimization aimed to amplify the inflammatory chemotaxis effect mediated by MaMs.

**Figure 7:**
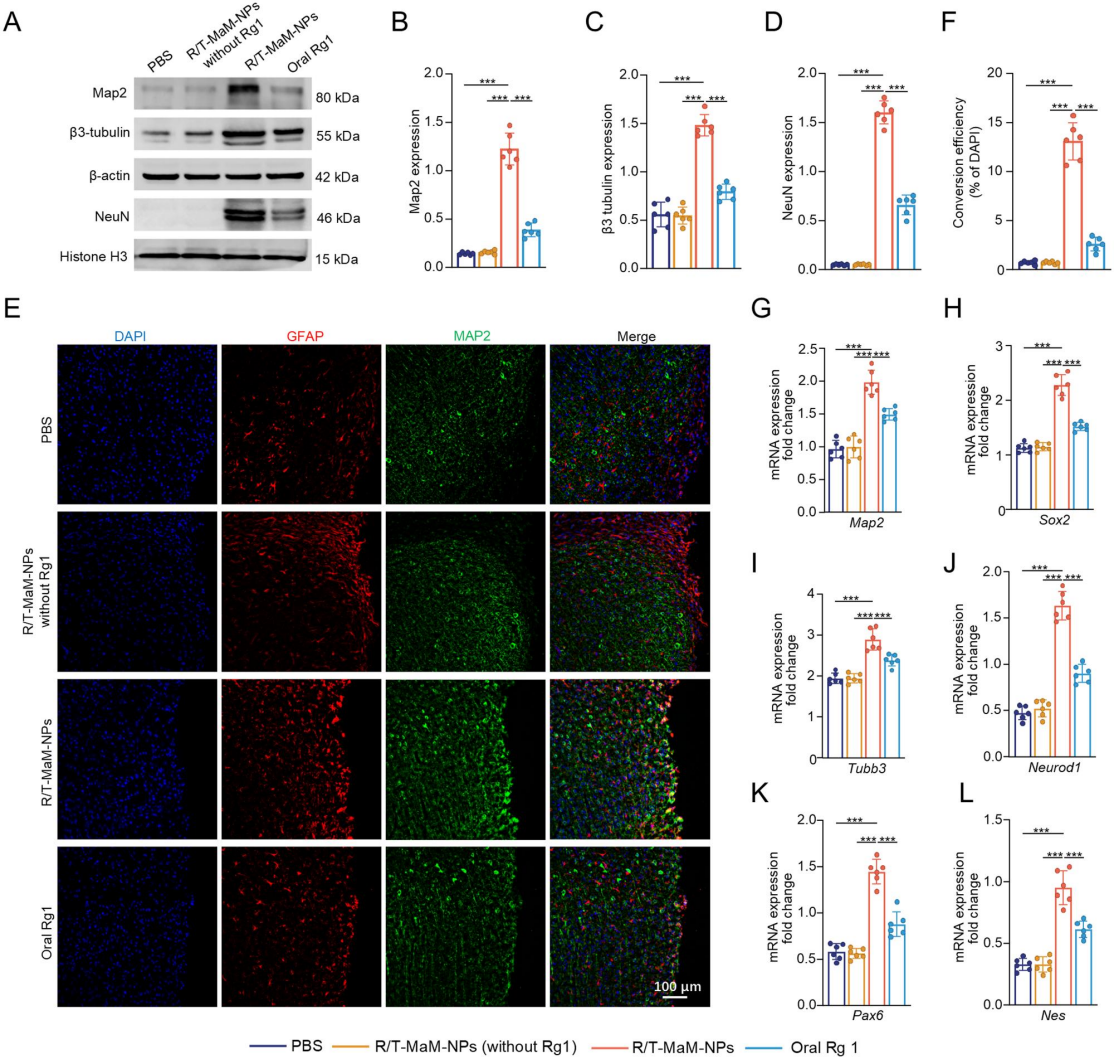
R/T-MaM-NPs promote astrocyte transdifferentiation within brain lesions *in vivo*. TBI mice were assigned to four groups: PBS, R/T-MaM‑NPs (without Rg1), R/T‑MaM‑NPs, and oral Rg1. (**A**) WB analysis of neuron‑associated proteins in RAs isolated from the injured cerebral hemisphere at 32 days post‑treatment. (**B–D**) Quantification of Map2, β3‑tubulin, and NeuN protein levels from (**A**). (**E**) Representative immunofluorescence images of Map2 expression in RAs from peri‑lesional sections across groups. Scale bar: 100 µm. (**F**) Transdifferentiation efficiency, quantified as the percentage of MAP‑positive RAs from (**E**). (**G–L**) Transcript levels of neuron‑associated genes in post‑TBI RAs measured by PCR. Data represent mean ± SD; (**B–D, F–L**) *n* = 6 (***P < 0.001).

Since the transdifferentiation of RAs is a long-term induction process, we administered a one-month treatment to TBI model mice with weekly supplementation of the nanoformulation. Brain samples were collected 32 days after treatment initiation. WB analysis of RAs from the injured cerebral hemisphere revealed that the R/T-MaM-NPs group displayed significant expression of mature neuronal proteins Map2, β3-tubulin, and NeuN, whereas the oral Rg1 group exhibited only minimal expression. In contrast, nearly no expression was detected in the PBS and empty carrier groups (Figure 7A–D). Subsequently, immunofluorescence staining of mouse brain sections was performed. Consistent with the WB results, RAs in the lesioned brain areas of R/T-MaM-NPs-treated mice exhibited substantial Map2 expression (transformation rate of 13%), whereas minimal expression was observed in other groups (Figure 7E and F). Furthermore, RNA sequencing was conducted on perilesional tissues from treated mice. PCA demonstrated spatial segregation of the R/T-MaM-NPs group from other groups, indicating distinct transcriptional profiles (Supplementary Figure S7A). GO analysis of enriched biological processes revealed that upregulated genes in R/T-MaM-NPs-treated lesioned tissues were positively correlated with nervous system development, neurogenesis, neuron generation, neuronal differentiation, and morphogenesis of differentiating neurons (Supplementary Figure S7B–D). To confirm RAs’ transdifferentiation at lesion sites post-R/T-MaM-NPs treatment, RAs were isolated from injured brain tissues via fluorescence-activated cell sorting. qRT-PCR analysis demonstrated the most significant upregulation of neuron-associated genes in R/T-MaM-NPs-treated RAs across all groups, including *Map2*, *Tubb3*, *Neurod1*, *Sox2*, *Pax6,* and *Nes* (Figure 7G–L). These findings align with *in vitro* RNA sequencing results.

Collectively, these findings provide compelling evidence that R/T-MaM-NPs treatment promotes the transdifferentiation of RAs into NLCs at the brain lesion site, playing a pivotal role in CNS repair. This therapeutic outcome critically hinges on our designed dual-peptide-modified biomimetic nanoformulation, which enables precise brain-targeted delivery of Rg1 across the BBB in post-TBI mice. However, both *in vitro* and *in vivo* evidence presented—primarily based on WB analysis, immunofluorescence colocalization, and *in vitro* patch-clamp recordings—reflect phenotypic observations of astrocytes toward a neuronal phenotype. The gold standard for confirming astrocyte-to-neuron transdifferentiation is genetic lineage tracing, and future studies will incorporate this approach to more rigorously validate cell fate conversion. Nevertheless, the current multi-dimensional evidence collectively supports that R/T-MaM-NPs can induce astrocytes to acquire neuron-like characteristics, laying a crucial foundation for subsequent in-depth investigations.

### R/T-MaM-NPs ameliorate the microenvironment in the cerebral lesion of TBI mice

**Figure 8:**
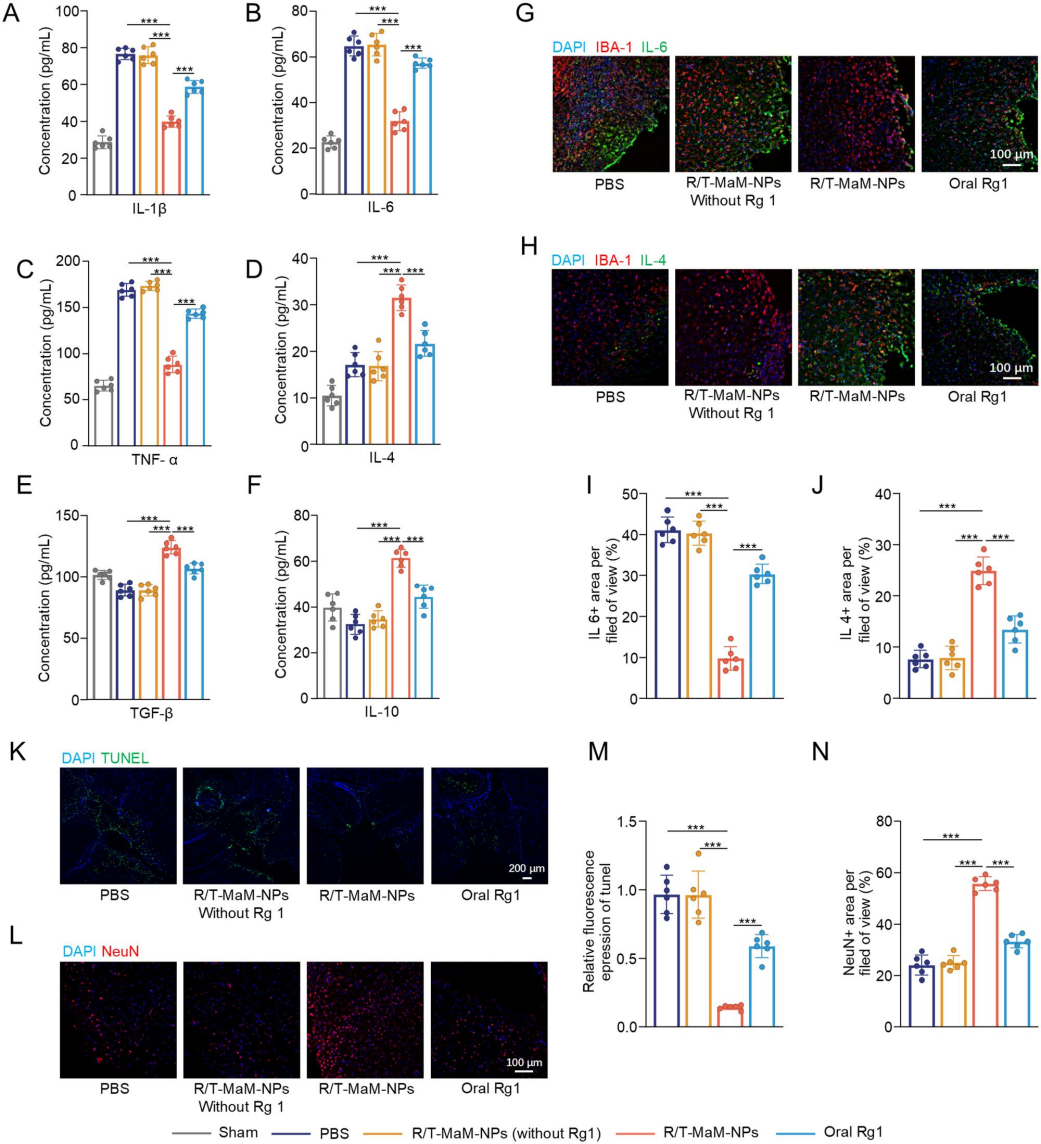
R/T-MaM-NPs ameliorate the microenvironment of cerebral lesions in TBI mice. (**A–F**) Levels of expression of IL-1β, IL-6, TNF-α, IL-4, TGF-β, and IL-10 in ipsilesional cerebral hemispheres-ELISA. (**G and H**) Representative immunofluorescence images showing the expression of IL‑6 and IL‑4 in peri‑lesional areas across treatment groups, scale bar: 100 μm, along with quantitative analysis of the percentage of positive cells (**I and J**). (**K**) TUNEL expression representative immunofluorescence images and (**M**) apoptotic cell ratio analysis. Scale bar: 200 μm. (**L**) Neuronal survival, representative immunofluorescence images, and (**N**) viable neuron quantification. Scale bar: 100 μm. Data are presented as mean ± SD; (**A–F, I, J, M, and N**) *n* = 6 (***P < 0.001).

It is well established that localized inflammatory storms following CNS injury exacerbate the lesional microenvironment [47, 48], and the quality of this microenvironment serves as a critical determinant for neuronal regeneration and repair. Given that R/T-MaM-NPs promote transdifferentiation of RAs at TBI lesion sites, we posit that this therapeutic strategy establishes a regeneration-permissive niche conducive to the maturation and integration of NLCs. To validate this hypothesis, brain tissue from mice 7 days after treatment was dissected, and cytokine levels in the injured area were measured by ELISA after homogenization. Post-TBI analysis revealed significant elevation of pro-inflammatory cytokines IL-1β, IL-6, and TNF-α, whereas anti-inflammatory mediators IL-4, TGF-β, and IL-10 showed no substantial increase. R/T-MaM-NPs treatment substantially reversed this inflammatory imbalance, demonstrating significant reductions in IL-1β, IL-6, and TNF-α expression compared to other groups, coupled with marked upregulation of IL-4, TGF-β, and IL-10 (Figure 8A–F). Oral Rg1 administration exhibited minimal therapeutic effects. Immunofluorescence staining of IL-6 and IL-4 corroborated the ELISA findings (Figure 8G–J), collectively indicating that R/T-MaM-NPs attenuated neuroinflammation at lesion sites, facilitating inflammatory resolution and functional recovery.

Additionally, we evaluated apoptosis and neuronal survival in perilesional brain tissues post-TBI. Immunostaining analysis demonstrated that the R/T-MaM-NPs group markedly suppressed cellular apoptosis in the area near the brain injury (Figure 8K and M), while concurrently preserving neuronal viability in injured brain tissues (Figure 8L and N). These findings collectively demonstrate that R/T-MaM-NPs remodel the post-TBI lesional microenvironment to create favorable conditions for post-injury neuronal regeneration and repair. In contrast, the oral Rg1 group exhibited negligible therapeutic efficacy. This further validates the superior drug delivery capabilities of our engineered brain-targeting biomimetic nanosystem.

### Therapeutic effects of R/T-MaM-NPs on TBI mice

**Figure 9:**
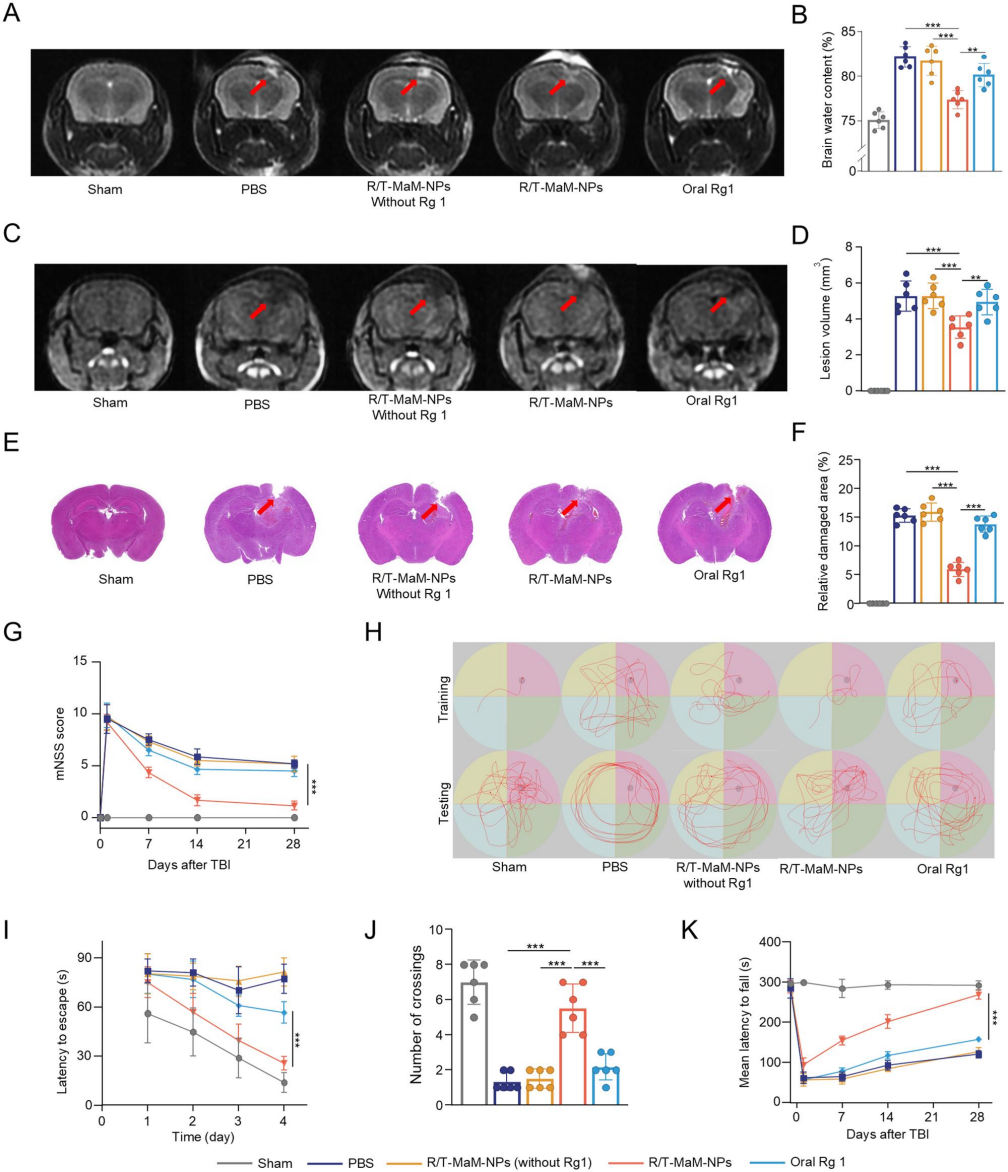
Nanoformulation therapy in TBI mice. (**A**) Representative T2WI on day 1 post‑treatment. (**B**) Quantification of cerebral edema in TBI mice assessed by brain water content via wet/dry weight measurement. (**C**) Representative T1WI at 32 days post‑treatment and (**D**) quantitative analysis of lesion volume. (**E**) H&E staining of brain tissue on day 32 post‑treatment. (**F**) Quantitative analysis of the damaged area from (**E**). (**G**) Modified Neurological Severity Score (mNSS) during the treatment period (higher scores indicate more severe injury). (**H**) Representative trajectory maps of Morris water maze training for each group at 28 days after treatment and representative trajectory maps of the test after training. (**I**) Analysis of escape latency during training. (**J**) Number of platform crossings during the probe trial. (**K**) Fall latency analysis in the rotarod test. Data are presented as mean ± SD; (**B, D, F, G, I–K**) *n* = 6 (**P < 0.01, ***P < 0.001).

Although the transdifferentiation-promoting and microenvironment-modulating effects of R/T-MaM-NPs have been well validated at the cellular level, the most direct and compelling evidence lies in the structural and functional therapeutic improvements observed in TBI mice. Disruption of the BBB following TBI often leads to cerebral edema, which subsequently elevates intracranial pressure and induces secondary injury to brain cells. We further evaluated the efficacy of R/T-MaM-NPs in alleviating cerebral edema. The extent of cerebral edema was first assessed *in vivo* across different treatment groups using MRI. As expected, T2DWI revealed smaller hyperintense areas in the R/T‑MaM‑NPs group compared to the TBI group (Figure 9A). Cerebral edema was further evaluated by measuring the wet‑to‑dry weight ratio of brain tissue. The results showed a significant increase in the wet‑to‑dry ratio in the TBI model group compared to the sham group, confirming the presence of pronounced edema. Compared with the TBI group, R/T‑MaM‑NPs‑treated TBI mice exhibited the lowest wet‑to‑dry ratio (Figure 9B). These findings indicate that R/T‑MaM‑NPs can alleviate post‑traumatic cerebral edema. Next, we examined the effect of R/T‑MaM‑NPs treatment on the cerebral lesion after TBI. T1DWI performed 32 days post‑treatment revealed that compared with untreated TBI mice, the lesion size in the R/T-MaM-NPs treatment group was significantly reduced by 32.7%, while the oral group only showed a 6.3% reduction (Figure 9C and D). H&E staining further demonstrated that R/T‑MaM‑NPs alleviated both the lesion area and tissue defects on day 32 post‑TBI (Figure 9E and F).

TBI can induce transient or permanent neurological dysfunction in cognitive, learning, memory, and motor domains, profoundly impacting post-TBI prognosis and rehabilitation [49]. Consequently, we conducted neurological function scoring and behavioral tests to investigate long-term recovery of neurological deficits. The mNSS assessment focused on impaired motor function, sensory acuity, and balance [50]. Results demonstrated that TBI-induced severe sensorimotor impairment, whereas R/T-MaM-NPs-treated mice demonstrated significantly greater reduction in neurological deficit scores over time (Figure 9G). Morris water maze testing performed at 28 days post-TBI revealed that R/T-MaM-NPs-treated mice exhibited significantly shorter escape latencies during training phases compared to other groups (Figure 9H and I). During the probe trial on day 5 (platform removed), these mice demonstrated increased platform crossings relative to controls (Figure 9H and J). Rotarod testing showed severe motor coordination deficits in TBI mice, whereas R/T-MaM-NPs treatment significantly prolonged fall latency compared to other treatment groups (Figure 9K). Collectively, these neurofunctional and behavioral improvements are primarily attributable to precision drug delivery by the R/T-MaM-NPs biomimetic nanosystem, enabling effective Rg1 accumulation at the brain lesion, and Rg1 improves the inflammatory microenvironment at the injury site after TBI in mice, provides a good soil for RAs transdifferentiation and CNS repair, and promotes the recovery of cognitive and motor functions in TBI mice.

In addition, nanoformulations circulate in the body for a long time after intravenous injection, so an ideal nanodrug delivery platform should have effective drug delivery and high biosafety. We evaluated major organs by tissue sections, and H&E staining images showed that after 32 days of injection, no significant tissue or structural damage was observed in the major organs of the mice in the different groups (Supplementary Figure S8), indicating the good long-term biosafety of the nanoformulation.

## Conclusions

In summary, the design of CNS-targeted nanodelivery systems necessitates three critical elements: (i) prolonged systemic circulation, (ii) efficient BBB penetration, and (iii) high-affinity cellular uptake by target cells. We developed R/T-MaM-NPs, a high-efficiency CNS delivery system demonstrating superior Rg1 brain biodistribution. This system synergistically combines MaM-mediated inflammatory tropism with dual-peptide targeting to efficiently penetrate the BBB and induce RAs transdifferentiation while ameliorating the post-TBI inflammatory milieu. These coordinated actions alleviated post‑TBI cerebral edema and promoted the recovery of neurocognitive and motor functions, contrasting sharply with the marginal therapeutic effects of oral Rg1 administration. Furthermore, this biomimetic nanostrategy exhibits broad applicability for CNS-impermeable therapeutics, including proteins, small molecules, and combinatorial regimens. The cell membrane-coating platform can be customized based on the pathomechanisms of various CNS disorders through: (i) selection of specialized membrane sources, and (ii) genetic engineering of tailored targeting ligands. Although short-term evaluations indicate that R/T-MaM-NPs exhibit an ideal safety profile, long-term biodistribution studies and chronic toxicity experiments remain necessary. This is one of the limitations of current nanodelivery strategies, and we will address these limitations in follow-up work. We believe this modular design paradigm opens new avenues for developing precision medicine strategies in neurological therapeutics.

## Supplementary Material

rbag059_Supplementary_Data

## Data Availability

All data required to evaluate the conclusions in the paper are available in the article itself and the Supplementary data.
