# Supplementary material for: Dual-peptide engineered macrophage membrane biomimetic nanosystem via targeting Rg1 delivery for traumatic brain injury therapy
Source: Regen Biomater. 2026 Mar 20;13:rbag059. doi: 10.1093/rb/rbag059 (PMC13106896; doi:10.1093/rb/rbag059)
Supplement: rbag059_Supplementary_Data [file rbag059_supplementary_data.docx]

**Supporting information**

**Dual-Peptide Engineered Macrophage Membrane Biomimetic Nano-System via Targeting Rg1 Delivery for Traumatic Brain Injury Therapy**

Weiquan Liao^1,2,^**^†^**, Zhichao Lu^1,2,^**^†^**, Ziheng Li^1,2,^**^†^**, Chenxing Wang^1,2^, Xingjia Zhu^1,2^, Jue Zhu^1,2^, Yongqi Zhu^1,2^, Jialiang Lin^1,2^, Jiajia Wen^1,2^, Xuanfeng Chen^1,2^, Jian Chen^1^, Jianhong Shen^1,^*, You Lang Zhou^2,^*, Peipei Gong^1,3,^*

1 Department of Neurosurgery, Affiliated Hospital of Nantong University, Medical School of Nantong University, Nantong, Jiangsu, 226001, China.

2 Research Center of Clinical Medicine, Affiliated Hospital of Nantong University, Nantong, Jiangsu, 226001, China.

3 Jiangsu Medical Innovation Center, Neurological Disease Diagnosis and Treatment Center, Affiliated Hospital of Nantong University, Nantong, Jiangsu, 226001, China

*** Corresponding address. E-mail:** **ntgpp@ntu.edu.cn (P.G.);** **youlangzhou@163.com (Y.L.Z.); tysjh@163.com (J.S.)**

**^†^** **These authors contributed equally to this work.**

**
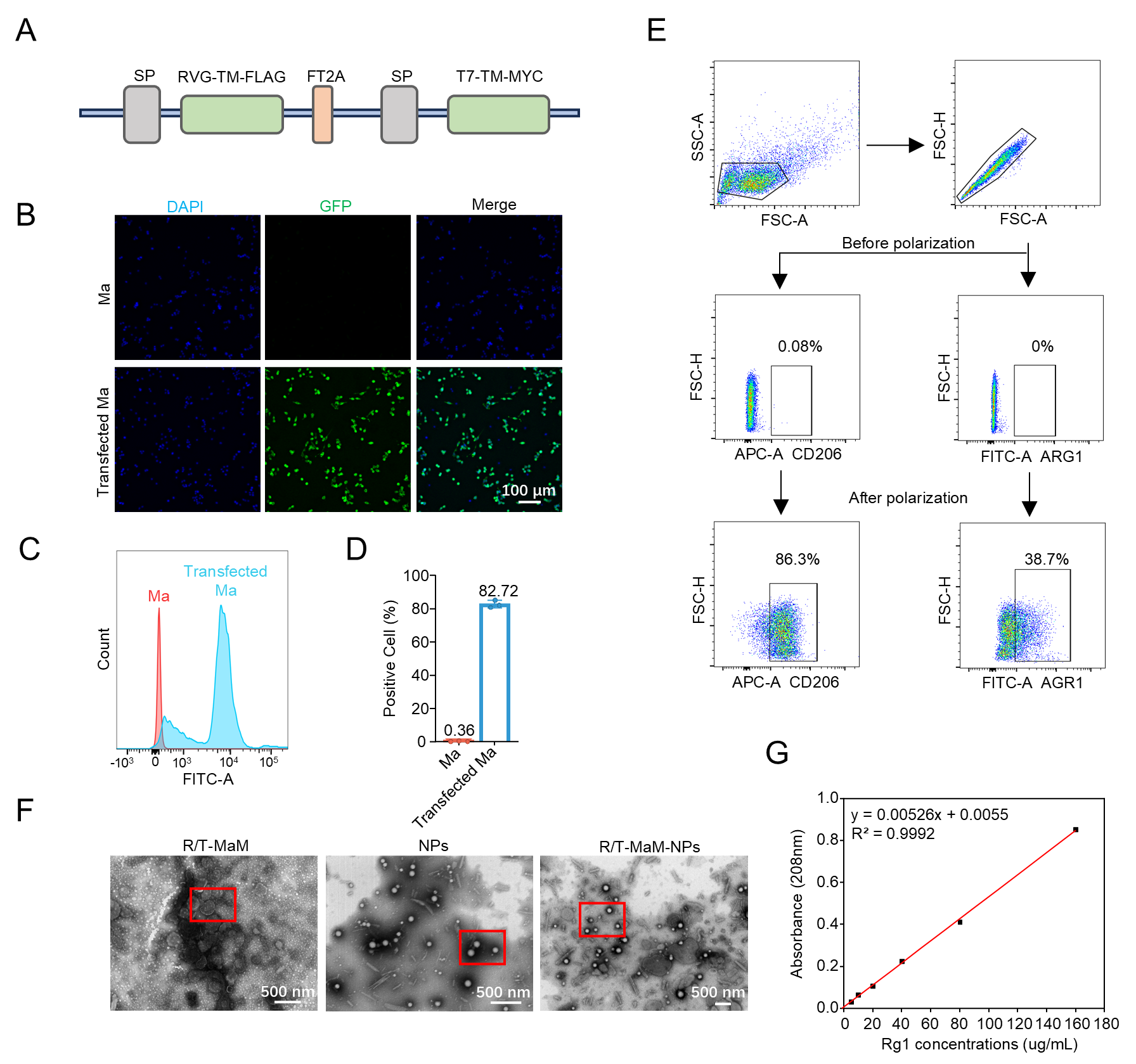
**

**Supplementary Figure S1** Preparation of nanoformulations. (A) Schematic of the lentiviral construct for RVG/T7 co‑expression: SP (signal peptide) and TM (transmembrane domain) ensure proper membrane localization; FLAG and MYC tags allow for detection and purification of the dual peptides; the FT2A linker containing a furin cleavage site facilitates proteolytic separation of the fusion protein under physiological conditions. (B) Representative fluorescence images of control lentivirus‑transduced RAW264.7 cells. Scale bar: 100 µm. (C) Flow cytometric analysis of lentiviral transduction efficiency. (D) Quantification of the flow cytometry data in (C). Results show that 86.81% of RAW264.7 cells expressed GFP 72 h post‑transduction. (E) Flow cytometry scatter plots showing M2‑type markers (CD206 and ARG1) in RAW264.7 cells after IL‑4 induction. (F) Low‑magnification TEM images of R/T‑MaM, NPs, and R/T‑MaM‑NPs. Scale bar: 500 nm. (G) Standard curve of Rg1 (208 nm). Data are presented as mean ±  standard deviation (SD); (D) n = 3.

**
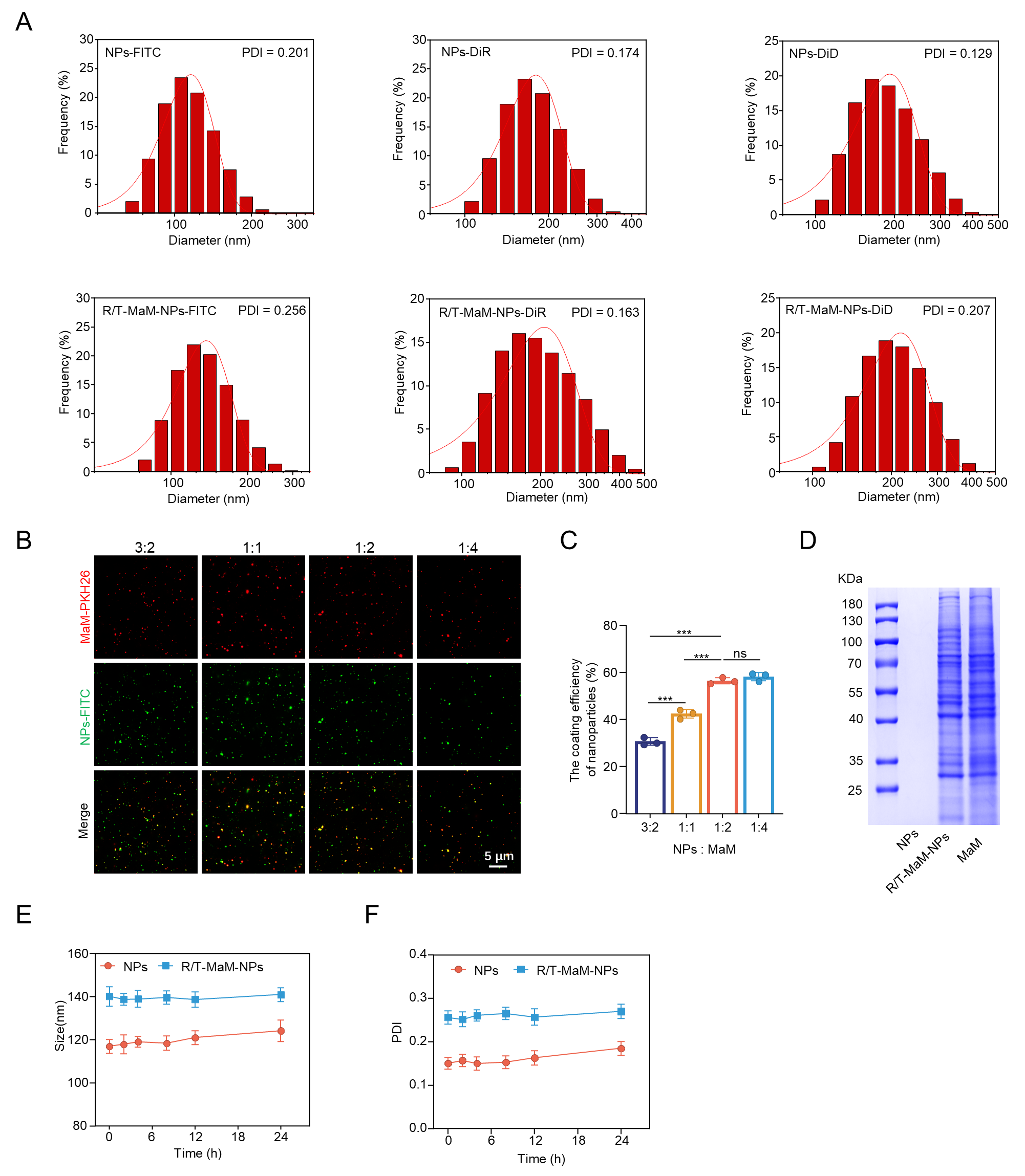
**

**Supplementary Figure S2** Characterization of nanoformulations. (A) Size distribution histograms of differently labeled nanoformulations. (B) Representative fluorescence images of R/T‑MaM‑NPs prepared with different formulations: FITC‑labeled NPs (green) and PKH26‑labeled MaM (red). Scale bar: 5 µm. (C) Quantitative analysis of fluorescence overlaps in (B) to assess the coating efficiency of the nanoformulations. (D) SDS‑PAGE analysis of the protein profiles in nanoformulations. (E, F) Plasma stability assessment of NPs and R/T‑MaM‑NPs over 48 h. Data are presented as mean ± SD; (C, E and F) n = 3 (***P < 0.001, ns, not significant).

**
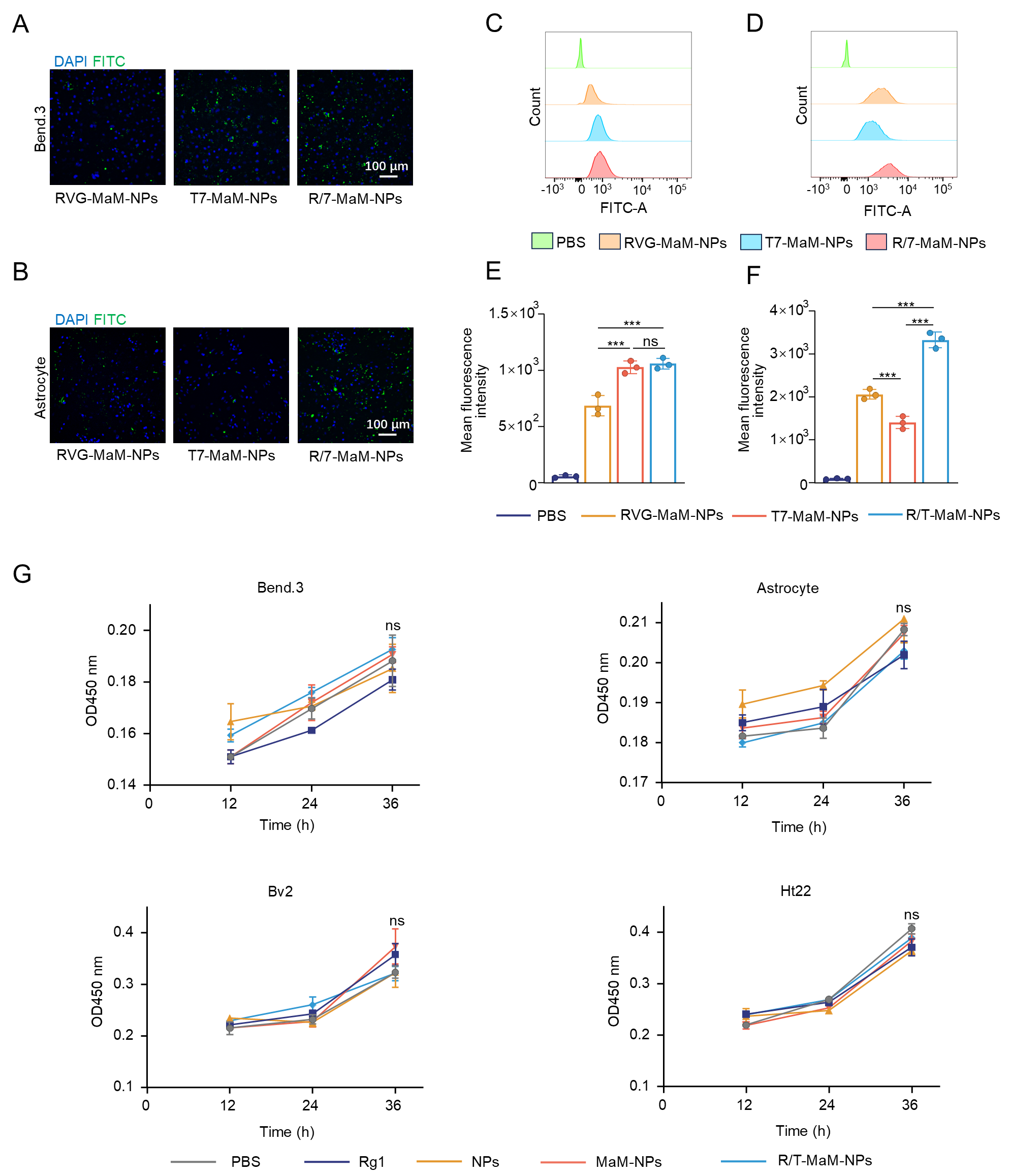
****Supplementary Figure S3** Cellular uptake and cytotoxicity assays of nanoformulations. (A and B) Representative immunofluorescence images showing the internalization of FITC‑labeled nanoformulations (green) by Bend.3 cells and primary astrocytes. Scale bar: 100 µm. (C and D) Flow cytometric analysis of the fluorescence intensity from Bend.3 cells and primary astrocytes that had taken up FITC‑labeled nanoformulations. (E and F) Quantification of fluorescence intensity from (C) and (D). (G) Cytotoxicity analysis of primary astrocytes, Bend.3, Bv2, and Ht22 cells exposed to different nanoformulations. Data are presented as mean ± SD; (E-J) n = 3 (***P < 0.001, ns, not significant).


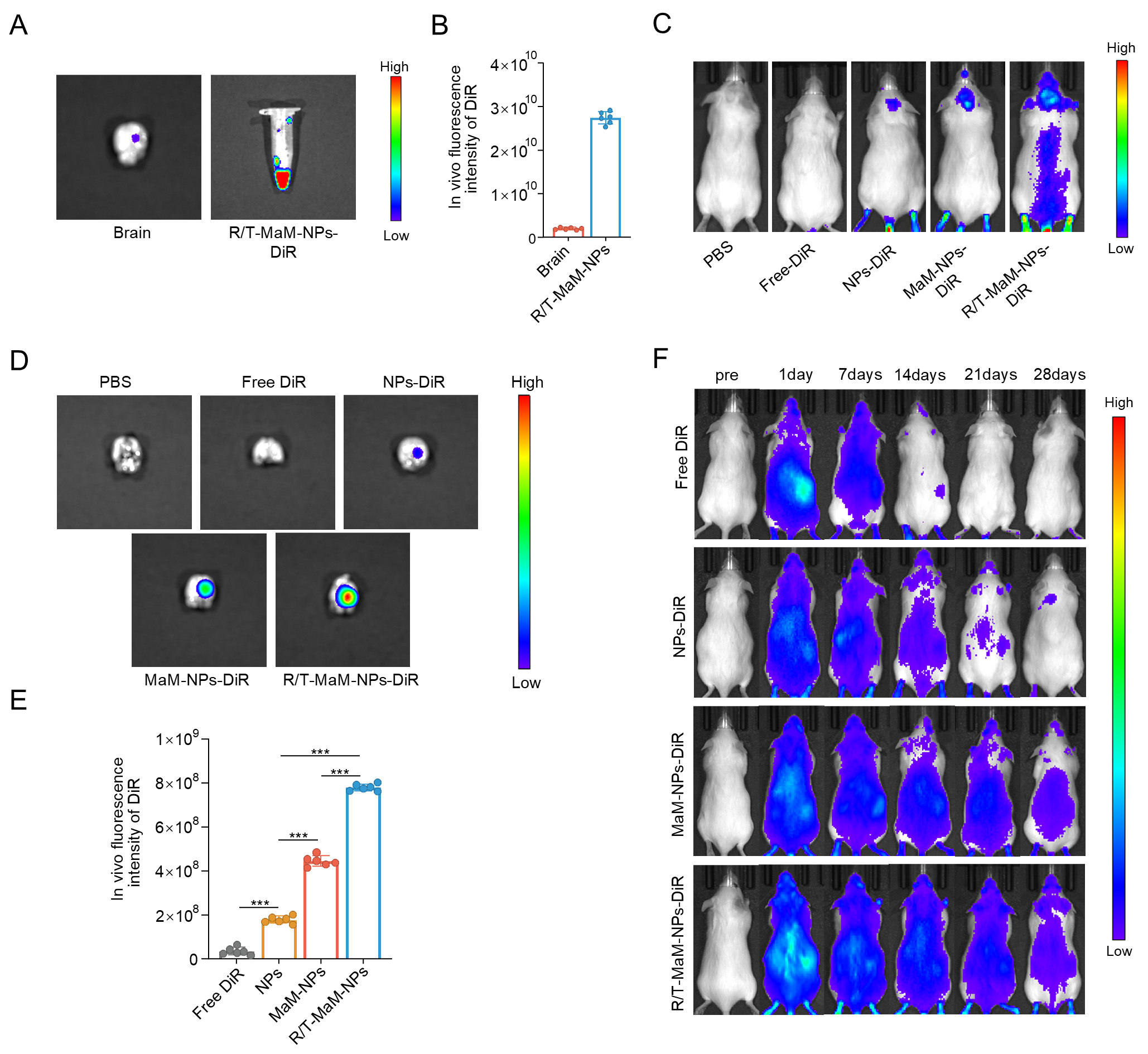
**Supplementary Figure S4** In vivo distribution and circulation time of nanoformulations. (A) Fluorescence signal comparison between the DiR‑labeled R/T‑MaM‑NPs solution prior to tail‑vein injection and the isolated brain tissue at the lesion site, measured by IVIS imaging. (B) Quantification of the fluorescence in (A). (C) Whole‑body biodistribution of TBI mice 32 days after injection of DiR‑labeled nanoformulations, acquired via IVIS imaging, alongside (D) a representative fluorescence image of the isolated brain. (E) Quantitative analysis of DiR signal intensity in the isolated brain from (D). (F) Representative whole‑body fluorescence images of healthy ICR mice at pre‑injection and 1, 7, 14, 21, and 28 days post‑tail‑vein injection of different DiR‑labeled nanoformulations, to evaluate their blood circulation time. Data are presented as mean ± SD; (B and E) n = 6 (***P < 0.001).

**
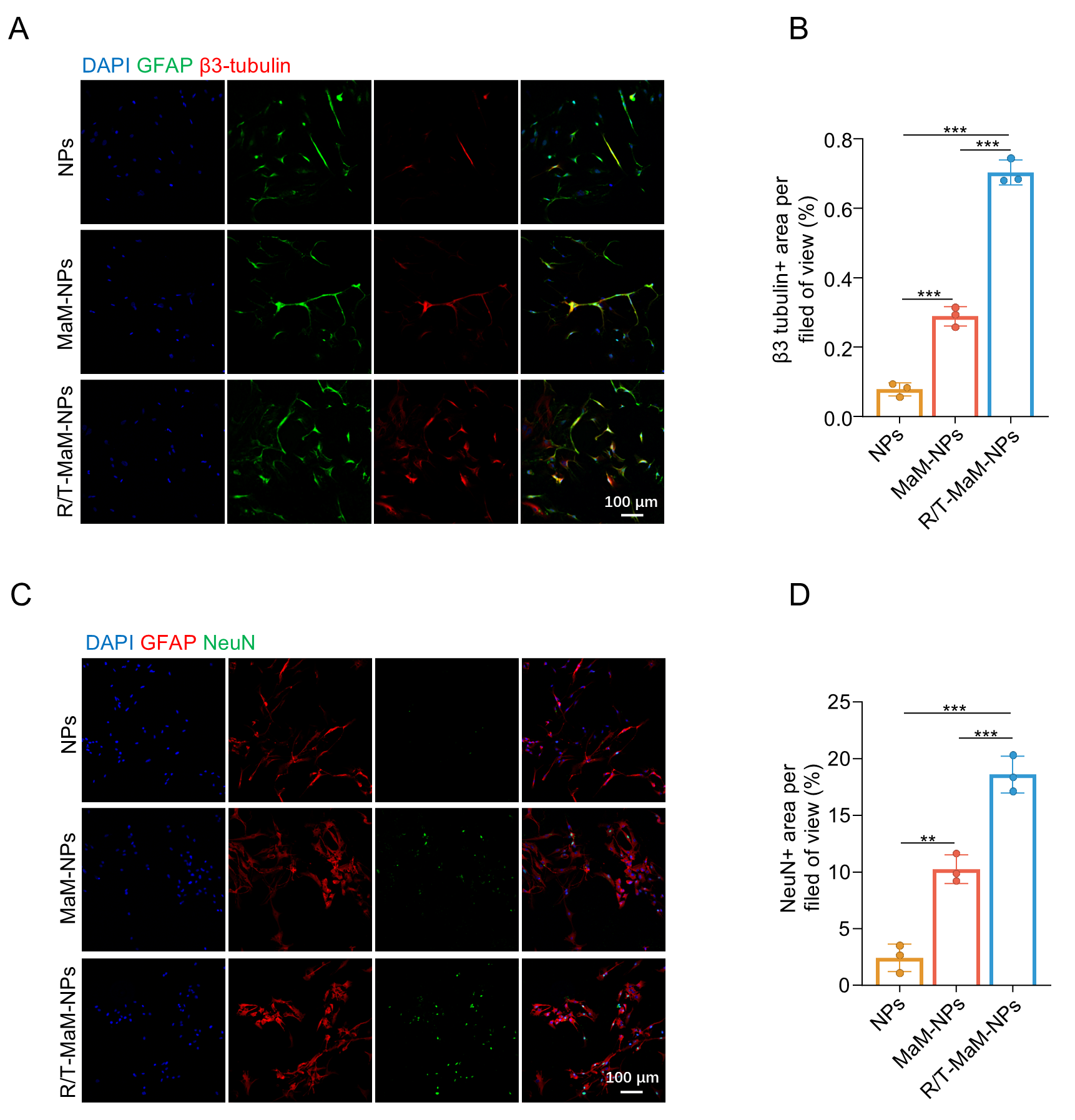
****Supplementary Figure S5** R/T-MaM-NPs induce transdifferentiation of RAs. (A and C) Representative immunofluorescence images of β3-tubulin and NeuN expression in RAs co-cultured with different nanoformulations in an in vitro BBB transwell model for 7 days. Scale bar: 100 μm. (B and D) Quantitative statistics of the proportion of RAs expressing β3-tubulin and NeuN in different groups from (A and C), respectively. Data represent mean ± SD, (B and D) n = 3 (**P < 0.01, ***P < 0.001).

**
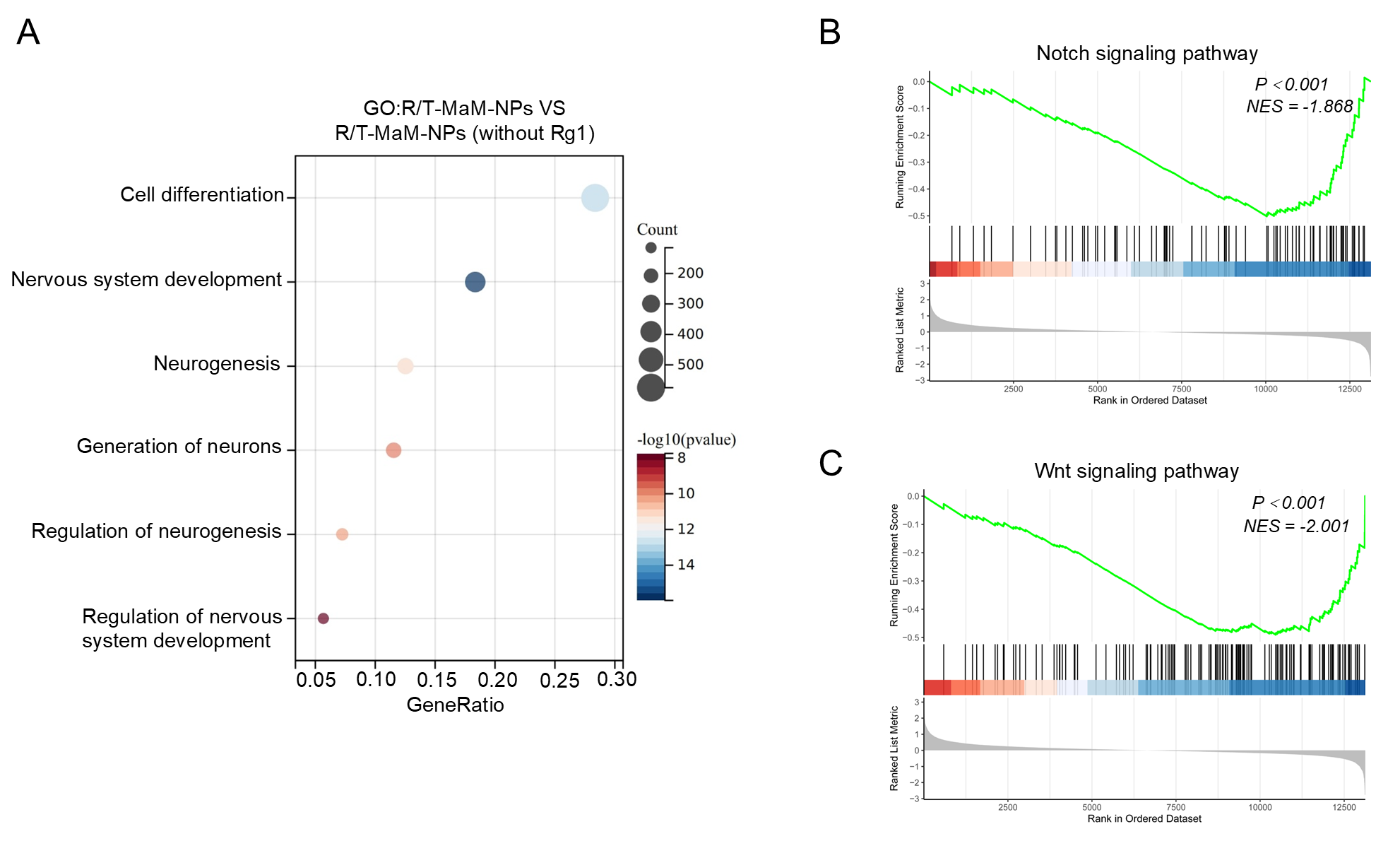
Supplementary Figure S6** RNA sequencing of RAs after 7 days of co-culture with different nanoformulations. (A) the Gene ontology (GO) analysis of the up-regulated genes in the R/7-MaM-NPs group compared to the R/7-MaM-NPs (without Rg1) group. (B and C) GSEA results of upregulated genes in R/T-MaM-NPs-treated RAs versus PBS controls. (A-C) n = 3 per group.


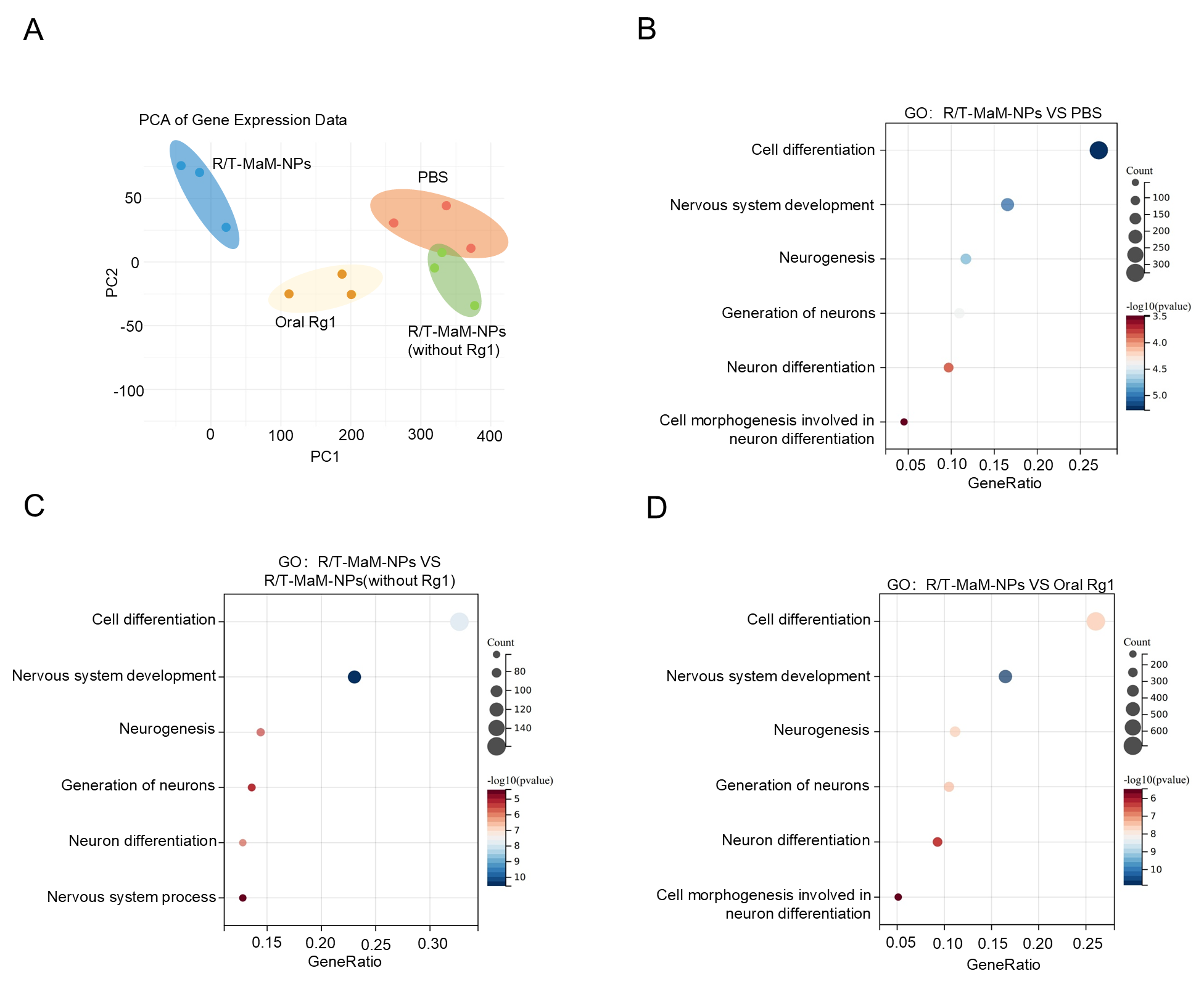
**Supplementary Figure S7** RNA sequencing of brain tissue from the injured area of TBI model mice after 32 days of treatment with different nanoformulations. (A) Principal component analysis (PCA) of RNA sequencing data from brain injury area tissues post-treatment. (B-D) GO analysis of up-regulated genes in the R/7-MaM-NPs group compared with the PBS, R/7-MaM-NPs (without Rg1) and Oral Rg1 groups. (A-D) n = 3 per group.

**
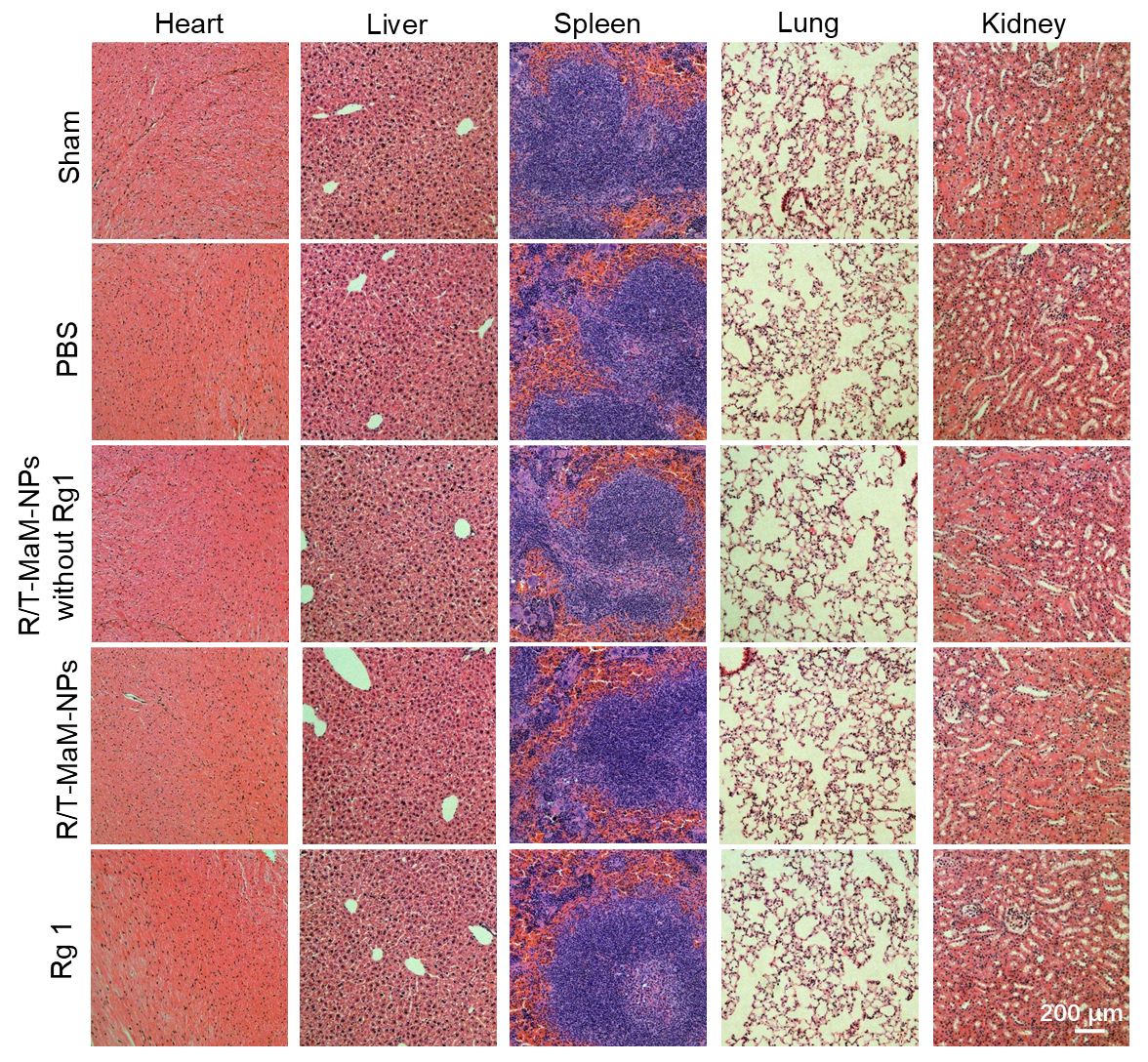
Supplementary Figure S8** Biosafety assessment of nanoformulations. Histological analysis of major organs (heart, liver, spleen, lung, kidney) from nanoformulation-treated mice. Scale bar: 200 μm, n = 6 per group.

**Supplementary Table S1** Antibodies used in this paper

|  | Antibodies | Company | Application |
| --- | --- | --- | --- |
| 1 | anti-MAP2 Polyclonal antibody (17490-1-AP) | Proteintech | WB/IF |
| 2 | anti-NeuN Monoclonal antibody (T55515M) | ab-mart | WB |
| 3 | anti-beta III Tubulin Polyclonal antibody (ab18207) | abcam | WB/IF |
| 4 | anti-Histone H3 Polyclonal antibody (17168-1-AP) | Proteintech | WB |
| 5 | anti-Beta Actin Monoclonal antibody (66009-1-Ig) | Proteintech | WB |
| 6 | anti-Myc-Tag (19C2) mAb (M20002S) | ab-mart | WB |
| 7 | anti-DYKDDDDK-Tag(3B9) mAb (M20008S) | ab-mart | WB |
| 8 | HRP labeled goat anti-mouse IgG(H+L) (A0216) | Beyotime | WB |
| 9 | HRP labeled goat anti-Rabbit IgG(H+L) (A0208) | Beyotime | WB |
| 10 | anti- Wnt3a Polyclonal antibody (26744-1-AP) | Proteintech | WB |
| 11 | anti- GSK3beta Polyclonal antibody (22104-1-AP) | Proteintech | WB |
| 12 | anti- Phospho-GSK3beta Polyclonal antibody (14850-1-AP) | Proteintech | WB |
| 13 | anti-Notch-1 Polyclonal antibody (20687-1-AP) | Proteintech | WB |
| 14 | anti-MYC tag Monoclonal antibody (60003-2-IG) | Proteintech | IF |
| 15 | anti-DYKDDDDK tag Polyclonal antibody (20543-1-AP) | Proteintech | IF |
| 16 | anti-IL-6 Monoclonal antibody (66146-1-Ig) | Proteintech | IF |
| 17 | anti-IL-4 Monoclonal antibody (66142-1-Ig) | Proteintech | IF |
| 18 | anti-NeuN Monoclonal antibody (NBP1-92693) | Novus Biologicals | IF |
| 19 | anti-Iba1 Polyclonal antibody (019-19741) | wako | IF |
| 20 | anti-GFAP Monoclonal antibody (60190-1-Ig) | Proteintech | IF |
| 21 | anti-GFAP Monoclonal antibody (R22776) | zenbio | IF |
| 22 | anti-Tyrosine Hydroxylase Monoclonal antibody (R381111) | zenbio | IF |
| 23 | anti-Choline Acetyltransferase Polyclonal antibody (AB144P) | Merck | IF |
| 24 | anti-VGLUT1 Polyclonal antibody(55491-1-AP) | Proteintech | IF |
| 25 | Donkey Anti-Goat IgG H&L (Alexa Fluor® 594) (ab150132） | abcam | IF |
| 26 | Donkey Anti-Mouse IgG H&L (Alexa Fluor® 488) (ab150105) | abcam | IF |
| 27 | Donkey Anti-Rabbit IgG H&L (Alexa Fluor® 488) (ab150073) | abcam | IF |
| 28 | Donkey Anti-Rabbit IgG H&L (Alexa Fluor® 555) (A-31572) | Invitrogen | IF |
| 29 | Anti-CD206 Monoclonal Antibody (MMR)APC (17-2061-82) | Invitrogen | Flow |
| 30 | Anti-Arginase 1 Monoclonal Antibody (Alexa Fluor® 488)  (53-3697-82) | Invitrogen | Flow |

**Supplementary Figure S2** PCR sequences

| **Gene** | **Forward primer sequence (5’-3’)** | **Reverse primer sequence (5’-3’)** |
| --- | --- | --- |
| *Map2* | GCTGTAGCAGTCCTGAAAGGTG | CTTCCTCCACTGTGGCTGTTTG |
| *Tubb3* | ATGAGGCCTCCTCTCACAAG | AGTTGTCGGGCCTGAATAGG |
| *Neurod1* | CACGCAGAAGGCAAGGTGTC | GTCATGTTTCCACTTCCTGTTGT |
| *Sox2* | AACGGCAGCTACAGCATGATGC | CGAGCTGGTCATGGAGTTGTAC |
| Pax6 | TTTAACCAAGGGCGGTGAGC | TCACTCCGCTGTGACTGTTC |
| *Nes* | AGGAGAAGCAGGGTCTACAGAG | AGTTCTCAGCCTCCAGCAGAGT |
| *Gapdh* | AGGTCGGTGTGAACGGATTT | TGCCGTGAGTGGAGTCATAC |

**Supplementary Table S3** Characterization of nanoparticles

| Nanoparticles (n = 3) | Size (nm) | PDI | Zeta potential (mV) |
| --- | --- | --- | --- |
| NPs | 116.6 ± 34.0 | 0.187 | -0.72 ± 5.44 |
| R/T-MaM-NPs | 141.9 ± 40.5 | 0.236 | -12.43 ± 13.50 |
| NPs-FITC | 115.9 ± 28.8 | 0.201 | -8.07 ± 10.50 |
| R/T-MaM-NPs-FITC | 138.1 ± 36.7 | 0.256 | -9.98 ± 6.64 |
| NPs-DiR | 179.3 ± 43.6 | 0.174 | 0.40 ± 6.70 |
| R/T-MaM-NPs-DiR | 202.6 ± 69.7 | 0.163 | -11.60 ± 8.72 |
| NPs-DiD | 191.8 ± 55.0 | 0.129 | -0.95 ± 8.23 |
| R/T-MaM-NPs-DiD | 212.5 ± 60.4 | 0.207 | -9.86 ± 7.12 |
